# The long-term impacts of opioid use before and after joint arthroplasty: matched cohort analysis of New Zealand linked register data

**DOI:** 10.1093/fampra/cmad112

**Published:** 2023-12-05

**Authors:** Yana Pryymachenko, Ross Wilson, John Haxby Abbott, Michelle Dowsey, Peter Choong

**Affiliations:** Department of Surgical Sciences, Centre for Musculoskeletal Outcomes Research, University of Otago, Dunedin, New Zealand; Department of Surgical Sciences, Centre for Musculoskeletal Outcomes Research, University of Otago, Dunedin, New Zealand; Department of Surgical Sciences, Centre for Musculoskeletal Outcomes Research, University of Otago, Dunedin, New Zealand; Department of Surgery, St Vincent’s Hospital, University of Melbourne, Melbourne, Australia; Department of Surgery, St Vincent’s Hospital, University of Melbourne, Melbourne, Australia

**Keywords:** opioids, joint replacement, osteoarthritis, cohort analysis, propensity score, New Zealand

## Abstract

**Background:**

Opioids are commonly used both before and after total joint arthroplasty (TJA).

**Objective:**

The objective of this study was to estimate the long-term effects of pre- and perioperative opioid use in patients undergoing TJA.

**Methods:**

We used linked population datasets to identify all (*n* =18,666) patients who had a publicly funded TJA in New Zealand between 2011 and 2013. We used propensity score matching to match individuals who used opioids either before surgery, during hospital stay, or immediately post-discharge with individuals who did not based on a comprehensive set of covariates. Regression analysis was used to estimate the effect of opioid use on health and socio-economic outcomes over 5 years.

**Results:**

Opioid use in the 3 months prior to surgery was associated with significant increases in healthcare utilization and costs (number of hospitalizations 6%, days spent in hospital 14.4%, opioid scripts dispensed 181%, and total healthcare costs 11%). Also increased were the rate of receiving social benefits (2 percentage points) and the rates of opioid overdose (0.5 percentage points) and mortality (3 percentage points). Opioid use during hospital stay or post-discharge was associated with increased long-term opioid use, but there was little evidence of other adverse effects.

**Conclusions:**

Opioid use before TJA is associated with significant negative health and economic consequences and should be limited. This has implications for opioid prescribing in primary care. There is little evidence that peri- or post-operative opioid use is associated with significant long-term detriments.

**Key messages**

- Opioid use before total joint arthroplasty is associated with long-term effects
- Strongest association for healthcare utilization and costs 5 years after surgery
- No association for peri- or post-operative opioid use
- Little difference among subgroups (ethnicity, sex, age, income)

## Introduction

Use of opioid medications for chronic pain associated with hip and knee osteoarthritis is discouraged by clinical practice guidelines,^1–3^ but remains common.^4–6^ Patients undergoing or awaiting total joint arthroplasty (TJA) for late-stage osteoarthritis typically have moderate to severe pain not adequately relieved by conservative treatments, and are therefore at higher risk of opioid prescribing.^5,7^ It has been reported that pre- and perioperative opioid use is associated with reduced pain relief following surgery and increased risk of surgical complications, early revision,^8–10^ and persistent post-operative opioid use (3–12 months after surgery),^4,8,11,12^ but there is little evidence regarding any longer-term impacts of pre- and perioperative opioid use.

Preoperative opioid prescribing may also have negative socioeconomic impacts. Some evidence suggests that preoperative opioid use is an influencing factor in lower return-to-work rates post-surgery.^13–16^ However, no studies to date have looked at broader economic, social, and healthcare outcomes following pre- and perioperative opioid use around TJA.

This paper examines the medium- to long-term health, economic, and social outcomes of pre- and perioperative opioid use in patients undergoing total hip and knee arthroplasty. Specifically, we aim to identify whether pre-operative opioid use (within 3 months prior to TJA), peri-operative use (in hospital following TJA), and immediate post-operative use (post-discharge, within 30 days of TJA) are associated with increased healthcare consumption, increased risk of adverse events, and worse socioeconomic outcomes over 5 years post-TJA. We make use of comprehensive linked national population datasets, providing a complete record of all TJAs performed in the New Zealand (NZ) public healthcare system and allowing for long-term follow-up and inclusion of a wide range of health and socioeconomic outcomes; and a propensity score matching approach using a large set of covariates to minimize potential confounding and support causal interpretation of our estimated effects.

## Methods

### Study cohort

The study cohort consists of all individuals who underwent publicly funded knee or hip arthroplasty in NZ (accounting for about 70% of all TJAs in NZ^17^) between June 2011 and December 2013 (to allow for a 5-year follow-up). We excluded individuals not residing in NZ throughout the one year before surgery (to facilitate complete identification of opioid use and baseline covariates); and those having TJA on another joint within 5 years of the index procedure (to avoid confounding post-surgery outcomes with those related to a subsequent surgery).

TJAs were identified using the Australian Refined-Diagnosis Related Groups classification, versions 6.0-7.0 (knee arthroplasty: I04A, I04B; hip arthroplasty: I03A, I03B). Opioid analgesics were identified from the NZ Pharmaceutical Management Agency (PHARMAC) Therapeutic Group Reference table, corresponding to ATC code N02A (Opioid Analgesics).

### Data sources

We used data from the Integrated Data Infrastructure (IDI), a linked research database maintained by Statistics NZ containing individual-level data covering the entire NZ population from government administrative datasets, Statistics NZ surveys, and non-governmental organizations.^18^ We used Ministry of Health hospital discharge records to identify all patients undergoing TJA in the public healthcare system and pharmaceutical dispensing records to measure opioid medication use before and after surgery. See the Supplementary Appendix for details of the datasets used to calculate covariates and outcomes.

### Treatment measures

We performed a separate analysis for each of the following treatment variables (each compared to their respective matched control groups without opioid dispensing over the corresponding period):

1 Pre-surgery opioid use, defined as being dispensed opioid medications at least once during the 3 months before surgery (***preoperative use***);
2 Opioid use during hospital stay, excluding intraoperative use (at least one dispensing) (***perioperative use***); and
3 Post-discharge opioid use within 30 days of surgery (at least one dispensing) (***post-discharge use***).

As these analyses were conducted independently, an individual could be in the treatment (opioid use) group in none, one, or more than one of the separate analyses.

### Outcomes

Multiple outcomes were considered to provide a comprehensive perspective on the potential impacts of preoperative, perioperative, and post-discharge opioid use (Box 1). In the primary analysis the outcomes were measured over a period of 5 years from the end of the baseline opioid use period (the day before surgery for preoperative use analysis, the day of discharge for perioperative use, and 30 days after surgery for post-discharge use). We also conducted trajectory analysis for some (of the more common) outcomes, which were measured during each of the 60 months of the 5-year period. Details and data sources for each of the outcomes analysed are presented in Supplementary Table A.1.

**Box 1: Outcome measures analysed**

**Healthcare utilization**

- Number of hospital admissions
- Days spent in hospital
- Number of emergency department visits
- Number of prescriptions dispensed
- Number of opioid prescriptions dispensed
- Opioid use (morphine equivalent daily average dose)

**Healthcare costs**

- Total public healthcare costs
- Hospitalization costs
- Outpatient and emergency department costs
- Pharmaceutical costs
- Laboratory testing costs
- Accident and injury compensation costs

**Socioeconomic outcomes**

- Total income
- Income from salary and wages
- Number of months in employment
- Receipt of any social welfare benefits
- Number of months receiving social welfare benefits
- Any criminal convictions

**Incidence of adverse events and health conditions**

- TJA revision surgery
- Death
- Injury insurance claims (motor vehicle accident, other injury)
- Opioid overdose
- Constipation
- Bowel obstruction
- Fracture
- Fall
- Myocardial infarction
- Heart failure

### Covariates

Use of the comprehensive linked population datasets allowed us to control for a wide range of important covariates that are potentially associated with pre- or perioperative opioid use as well as the outcomes.^11^ The covariates included demographic and geographic characteristics, prior health service use, socioeconomic variables, surgery characteristics, and comorbid health conditions. For analysis of perioperative and post-discharge opioid use, preoperative use was included as a covariate; for analysis of post-discharge opioid use, perioperative opioid use was also included as a covariate. The full details on the covariates used and their construction can be found in Supplementary Table A.3.

### Matching

The matching process and results have been described previously.^19^ In brief, matching was performed separately for each treatment of interest, using a combination of exact matching on key variables (sex, Māori ethnicity [NZ’s indigenous population], year of surgery, and type of surgery) and propensity score matching on all remaining covariates, using 1:1 nearest neighbour matching with replacement. The matching process yielded excellent balance on all covariates (Supplementary Table A.4), with no unmatched treatment observations for the perioperative and post-discharge use groups, and <0.5% unmatched for the preoperative use group.

### Outcome analysis

For our primary analysis, we used a linear regression model where we regressed the outcome of interest (measured over a 5-year period) on the treatment variable and covariates. The regressions were adjusted for baseline covariates and the pre-treatment measure of the outcome. As matching with replacement was used for all 3 treatment variables, weighted regression was used to obtain appropriate estimates of the average treatment effect. Confidence intervals, at the 95% confidence level, were calculated using cluster-robust standard errors to account for the matched pairs generated by the matching process.^20^ In addition, we analysed the trajectory of key outcomes over the follow-up period by regressing the outcome of interest (measured during each month of the 5-year period) on the month and the interaction between the month and the treatment status.

Separate sub-group analyses were conducted by ethnicity (Māori and non-Māori patients), type of surgery (hip and knee arthroplasty) and sex to investigate between-group differences.

### Sensitivity analysis

To check the robustness of our results to model specification, we estimated the regressions using no covariates and using all of the covariates included in the matching process. Because the matching process should result in a close balance of covariates between the treatment and control groups, the results should not be sensitive to the specification of regression model.^21^ Second, we checked whether the results are sensitive to the choice of estimation model. We estimated the results using Poisson regression for continuous outcomes and using logistic regression for binary outcomes. Finally, we performed the analysis for post-discharge use analysis using an alternative matching model (which sets a calliper of 0.25 SD of the propensity score; this model yielded better balance but resulted in 1% unmatched treatment observations (see the matching report^19^)).

### Software

The analyses were undertaken in R (version 3.6) in the Statistics NZ IDI data lab.^22^ The R package MatchIt^23^ was used to perform the matching procedure.

The statistical analysis plan was pre-specified and published on the Open Science Framework^24^, and the results of the matching process were published prior to conducting the outcome analysis.^19^

## Results

### Characteristics of the study cohort

We identified 18,666 individuals with TJA during the study period, of whom 6,702 (36%) were dispensed opioids in the preoperative period, 8,967 (48%) in the perioperative period, and 7,812 (42%) in the post-discharge period. After matching, the treatment groups consisted of 6,702 individuals with preoperative opioid use, 8,967 individuals with perioperative use, and 7,812 with post-discharge use (with the same number of individuals in the respective control groups). Table 1 describes the baseline characteristics of the treatment and control cohorts after matching.

**Table 1.** Baseline characteristics of individuals undergoing total joint arthroplasty in New Zealand (2011–2013), after matching (means (SD) unless stated otherwise).

Variable	Preoperative opioid use				Perioperative opioid use				Post-discharge opioid use			
Observations, *n*	Treatment		Control		Treatment		Control		Treatment		Control	
	6,702		6,702		8,967		8,967		7,812		7,812	
**Demographic characteristics and geography**												
Age at surgery, years	69.4	(11.9)	70.1	(11.2)	67.7	(11.1)	67.8	(11.5)	69.5	(11.6)	69.1	(11.8)
Female, *n* (%)	4,014	(59.9)	4,014	(59.8)	4,836	(53.9)	4,836	(53.9)	4,350	(55.7)	4,350	(55.7)
Māori, *n* (%)	690	(10.3)	690	(10.3)	918	(10.2)	918	(10.2)	834	(10.7)	834	(10.7)
Urban residence, *n* (%)	5,655	(84.3)	5,775	(86.2)	7,461	(83.2)	7,455	(83.1)	6,588	(84.4)	6,573	(84.1)
**Health service use (prior to the treatment period)**												
Number of hospitalizations	0.57	(1.2)	0.53	(0.9)	0.53	(1.2)	0.51	(1.0)	0.66	(1.4)	0.64	(1.2)
Number of emergency department visits	0.44	(1.1)	0.46	(1.1)	0.38	(1.0)	0.34	(0.9)	0.51	(1.2)	0.45	(1.0)
Number of prescriptions dispensed (incl. opioids)	54.9	(83.1)	46.57	(56.7)	49.23	(91.5)	47.67	(68.7)	65.65	(104.8)	62.97	(87.5)
Opioid prescriptions in mg of OMEDD	13.8	(42.4)	7.73	(17.3)	7.04	(29.9)	7.35	(43.0)	10.81	(40.0)	6.90	(19.9)
Healthcare costs (total), NZD	6,599	(15 169)	6,227	(12 607)	6,134	(13 879)	5,936	(11 607)	7,725	(16 870)	7,508	(14 328)
**Socio-economic variables (prior to the treatment period)**												
Total income, 1,000 NZD	16.4	(15.0)	15.65	(15.3)	23.96	(25.9)	22.99	(22.0)	21.85	(21.0)	21.74	(21.7)
Number of months in employment, *n* (%)	1,815	(27.1)	1,674	(25.0)	2,904	(32.4)	2,784	(31.0)	2,046	(26.2)	2,097	(26.8)
Received social benefits, *n* (%)	783	(11.7)	741	(11.1)	972	(10.8)	1,092	(12.2)	960	(12.3)	927	(11.9)
Education (“No qualification” baseline), *n* (%)	2,124	(31.7)	2,163	(32.3)	2,814	(31.4)	2,943	(32.8)	2,472	(31.7)	2,547	(32.6)
Secondary school	1,485	(22.1)	1,476	(22.0)	2,061	(23.0)	2,016	(22.5)	1,716	(22.0)	1,713	(21.9)
Post-secondary education	1,206	(18.0)	1,224	(18.3)	1,758	(19.6)	1,776	(19.8)	1,425	(18.2)	1,497	(19.2)
Tertiary education	366	(5.5)	336	(5.0)	618	(6.9)	630	(7.0)	471	(6.0)	447	(5.7)
Unknown education	1,521	(22.7)	1,506	(22.5)	1,719	(19.2)	1,608	(17.9)	1,734	(22.2)	1,605	(20.5)
**Surgery characteristics**												
Length of stay, days	5.74	(4.2)	5.39	(3.3)	5.12	(3.1)	5.26	(4.5)	5.34	(2.8)	5.76	(4.8)
Knee surgery, *n* (%)	2,373	(35.4)	2,373	(35.4)	4,446	(49.6)	4,446	(49.6)	3,999	(51.2)	3,999	(51.2)
Discharged to another health facility, *n* (%)	984	(14.7)	945	(14.1)	222	(2.5)	1,479	(16.5)	1,191	(15.3)	1,011	(12.9)
**Comorbidities (prior to the treatment period)**, ***n* (%)**												
Depression	2,070	(30.9)	2,142	(32.0)	2,058	(22.9)	2,100	(23.4)	2,277	(29.2)	2,214	(28.3)
Anxiety	516	(7.7)	531	(7.9)	579	(6.5)	573	(6.4)	630	(8.1)	576	(7.4)
Hypertension/IHD/angina	2,832	(42.2)	2,937	(43.8)	3,678	(41.0)	3,900	(43.5)	3,639	(46.6)	3,648	(46.7)
Hyperlipidaemia	2,553	(38.1)	2,547	(38.0)	3,288	(36.7)	3,483	(38.8)	3,204	(41.0)	3,387	(43.4)
Antiplatelet agents	2,487	(37.1)	2,721	(40.6)	3,117	(34.7)	3,201	(35.7)	3,144	(40.3)	3,162	(40.5)
Congestive heart failure	3,234	(48.2)	3,225	(48.1)	3,987	(44.4)	4,089	(45.6)	3,879	(49.7)	3,912	(50.1)
Diabetes	861	(12.8)	897	(13.4)	1,140	(12.7)	1,188	(13.2)	1,239	(15.9)	1,377	(17.6)
Gastric acid disorder	3,468	(51.7)	3,534	(52.7)	4,092	(45.6)	4,161	(46.4)	3,972	(50.9)	3,999	(51.2)
Liver disease	849	(12.7)	915	(13.7)	903	(10.1)	888	(9.9)	1,074	(13.8)	939	(12.0)
Gout	624	(9.3)	558	(8.3)	783	(8.7)	837	(9.3)	756	(9.7)	807	(10.3)

NZD = New Zealand dollar; OMEDD = Oral morphine equivalent daily dose.

### Regression analysis

**Fig. 1.**
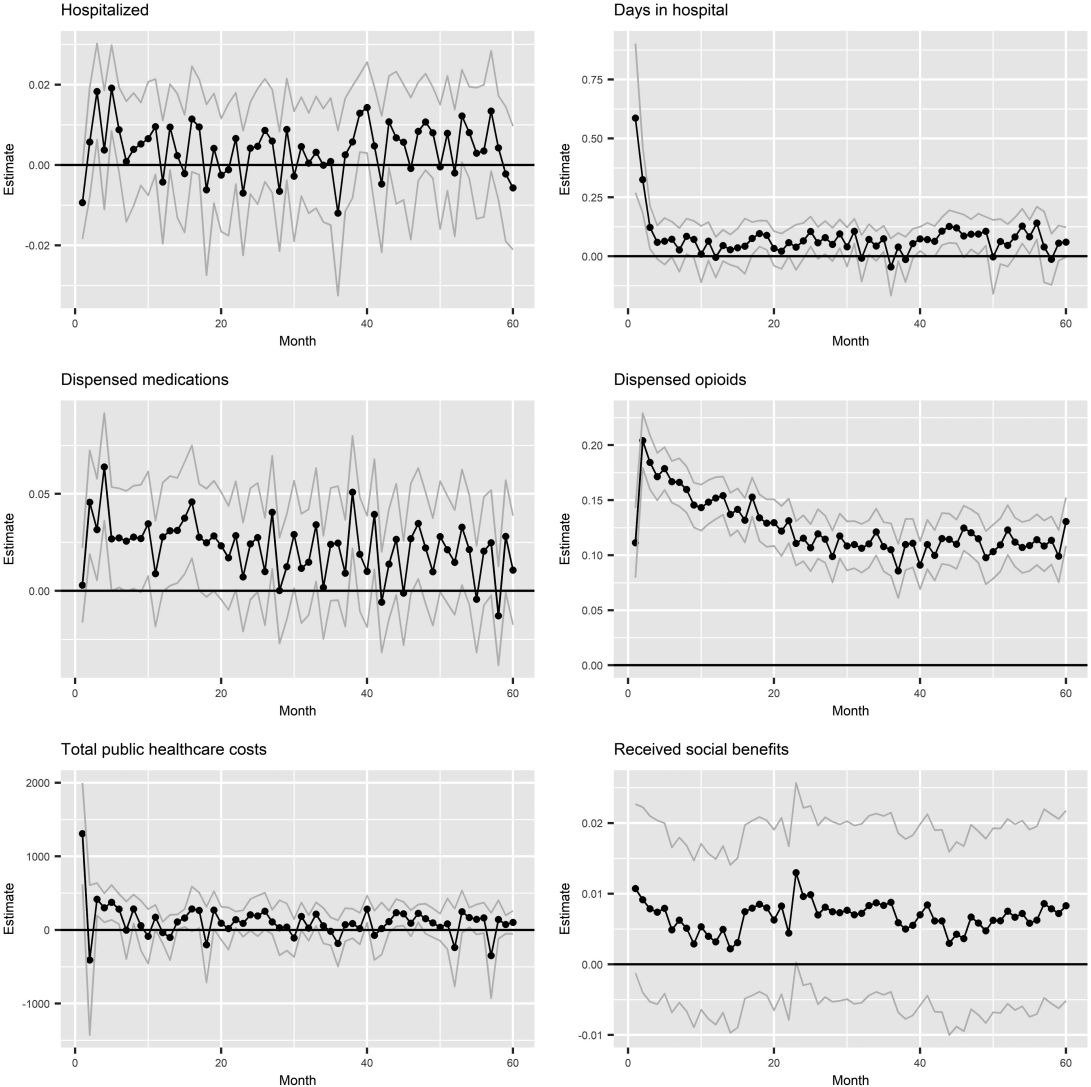
Trajectories of effect estimates for preoperative opioid use. Note: The grey lines represent the 95% confidence intervals.

Use of opioids during the 3 months before surgery (preoperative use) was associated with a significant increase in hospitalizations, days spent in hospital, opioid and total pharmaceutical use, and healthcare costs over 5 years post-surgery (Table 2). It was also associated with a higher probability of death, opioid overdose, and receipt of social welfare benefits. Other socioeconomic outcomes had the expected direction of association—preoperative opioid use being associated with worse outcomes—but were not statistically significant. Figure 1 presents estimates of these associations by month (for the outcomes with significant associations in Table 2). The associations with days in hospital and total healthcare costs were highest during the first month post-surgery (which includes the impact of longer length of stay in the index (surgery) admission), after which they were evenly distributed across the period. The association with opioid use was highest during the second month post-surgery, after which it gradually declined and stabilized at half of its original size at around 24-month mark. The associations for the rest of the outcomes were approximately stable across the 5-year period.

**Table 2.** Association between opioid use and long-term outcomes of total joint arthroplasty patients in New Zealand (2011–2013).

Outcome	Preoperative opioid use (*n* = 13,404)		Perioperative opioid use (*n* = 17,934)		Post-discharge opioid use (*n* = 15,624)	
	Est.	(95% CI)	Est.	(95% CI)	Est.	(95% CI)
**Healthcare utilization and healthcare costs**						
Hospitalizations	0.24	(0.03 to 0.45)	−0.10	(−0.27 to 0.08)	0.04	(−0.18 to 0.27)
Days in hospital	2.41	(0.96 to 3.85)	−1.51	(−2.35 to −0.66)	−1.42	(−2.84 to −0.01)
Emergency department visits	0.24	(−0.01 to 0.49)	−0.06	(−0.20 to 0.08)	0.06	(−0.17 to 0.28)
Prescriptions dispensed	50.94	(32.15 to 69.73)	1.28	(−8.48 to 11.03)	22.05	(7.39 to 36.71)
Opioid prescriptions (number)	19.67	(16.85 to 22.48)	3.27	(1.29 to 5.26)	13.41	(11.02 to 15.79)
Opioid prescriptions (OMEDD)	5.75	(4.27 to 7.24)	0.54	(−1.18 to 2.26)	4.22	(3.19 to 5.26)
Healthcare costs (total)	5 602	(2 652 to 8 552)	−3 109	(−5 489 to −730)	−2 932	(−6 133 to 270)
Outpatient (NNPAC) costs	917.6	(206.7 to 1 629)	55.06	(−602.3 to 712.4)	−184.8	(−897.6 to 528.1)
Lab costs	12.61	(5.06 to 20.17)	6.61	(1.71 to 11.51)	3.19	(−4.04 to 10.42)
Pharmaceutical costs	296.0	(−179.1 to 771.2)	−217.3	(−609.0 to 174.5)	101.6	(−473.3 to 676.6)
Hospitalization costs	4 194	(1 504 to 6 884)	−2 903	(−4 982 to −823)	−2 756	(−5 519 to 7)
Accident and injury costs	176.3	(−527.5 to 880.0)	598.2	(121.8 to 1 075)	393.4	(−190.2 to 976.9)
**Socio-economic outcomes**						
Income (total)	−2 137	(−5 200 to 927)	1 307	(−1 722 to 4 336)	202.3	(−2 611 to 3 015)
Income (wage and salary)	−402.8	(−3 205 to 2 399)	−893.0	(−3 755 to 1 969)	−958.4	(−3 538 to 1 621)
Months on benefits	0.34	(−0.19 to 0.88)	−0.19	(−0.55 to 0.18)	0.21	(−0.22 to 0.64)
Months of employment	−0.33	(−1.07 to 0.41)	0.12	(−0.43 to 0.66)	−0.29	(−0.81 to 0.23)
Received benefits	0.02	(0.01 to 0.03)	0.00	(−0.01 to 0.01)	0.01	(0.00 to 0.02)
Convicted	0.00	(0.00 to 0.01)	0.00	(−0.01 to 0.00)	0.00	(0.00 to 0.01)
**Incidence of adverse events/health conditions**						
TJA revision	0.01	(0.00 to 0.02)	0.00	(−0.02 to 0.17)	0.00	(0.00 to 0.01)
Deceased	0.03	(0.01 to 0.06)	−0.03	(−0.001 to 0.003)	−0.01	(−0.02 to 0.01)
Road accident	0.00	(−0.01 to 0.01)	0.00	(−0.02 to 0.00)	0.00	(0.00 to 0.01)
Other injuries	0.15	(−0.03 to 0.33)	0.07	(−0.01 to 0.00)	0.19	(0.06 to 0.31)
Overdose	0.005	(0.003 to 0.008)	0.001	(−0.03 to 0.00)	0.001	(−0.002 to 0.003)
Constipation	0.00	(−0.02 to 0.02)	−0.01	(−0.02 to 0.00)	−0.01	(−0.02 to 0.01)
Bowel obstruction	0.00	(−0.01 to 0.01)	0.00	(−0.01 to 0.00)	0.00	(0.00 to 0.01)
Fracture	0.00	(−0.02 to 0.03)	−0.02	(−0.01 to 0.00)	−0.02	(−0.04 to −0.01)
Falls	0.01	(−0.01 to 0.04)	−0.01	(−0.02 to 0.17)	−0.01	(−0.03 to 0.00)
Myocardial infarction	0.00	(−0.01 to 0.02)	0.00	(−0.001 to 0.003)	0.00	(−0.01 to 0.01)
Heart failure	0.00	(−0.02 to 0.02)	−0.01	(−0.02 to 0.00)	0.00	(−0.02 to 0.01)

Each estimate represents the result from a separate regression. All regressions are adjusted for baseline covariates (age, sex, ethnicity, education level, geography, comorbidities, year of surgery, and type of surgery) and the pre-treatment value of the outcome.

NNPAC = National Non-Admitted Patient Collection; OMEDD = Oral morphine equivalent daily dose; TJA = total joint arthroplasty.

For opioid use post-surgery, the results were somewhat mixed. Individuals taking opioids during the hospital stay (perioperative use) were dispensed more opioid prescriptions and had higher costs associated with accidents or injuries during the following 5 years than individuals not taking opioids during the hospital stay. However, they spent fewer days in hospital and had lower hospitalization costs. They were also less likely to die or have fractures. As seen in Supplementary Figure A.1, the associations with days in hospital and total healthcare costs were evenly distributed across the period, while the association with opioid dispensing was negative during the first month after discharge, spiked during the second month and stabilized during the next few months.

Individuals taking opioids post-discharge but within 30 days of surgery (post-discharge use) were dispensed more prescription medications (total and opioid medications) and were more likely to suffer a non-traffic accident or injury during following 5 years. They were less likely to have a fracture. The associations with both total dispensing and opioid dispensing were the highest during the first month after the post-discharge period (Supplementary Figure A.2).

Sensitivity analyses showed that the primary results reported in Table 2 were robust to the choice of matching model, model specification, and estimation model (Supplementary Tables A.5–A.10).

### Subgroup analysis

The results by sub-groups are presented in Supplementary Tables A.11–A.19. There were only minor differences between sub-groups for most outcomes.

## Discussion

Using opioids during the 3 months prior to surgery was associated with increased hospitalizations, prescriptions, including opioid prescriptions, and healthcare costs, and with increased risk of opioid overdose and all-cause mortality, over 5-year follow-up. These findings add weight to existing guideline recommendations discouraging opioid prescribing for patients awaiting hip and knee arthroplasty.^1–3^

Several methodological strengths have allowed this study to address significant gaps in prior knowledge regarding the long-term health, social and economic impacts of opioid use around TJA. The use of a comprehensive dataset covering the entire NZ population offers several advantages. First, we were able to analyse all publicly funded TJAs conducted in NZ during 2011–2013, allowing for generalizability of the results to this population. Second, we were able to control for an extensive set of covariates, follow-up individuals for 5 years post-surgery, and analyse a large set of outcomes, which have not been possible in previous studies. We were able to use pharmaceutical dispensing as our measure of opioid use rather than prescriptions or patient recall, which are less likely to reflect actual opioid use. A significant strength of this study is the research design: our comprehensive matching technique, with excellent between-group balance achieved across a large set of potential confounders, helps support causal interpretation of our results. Importantly, as prior opioid use (before the 3-month preoperative period) was included as a baseline covariate in all analyses, our results speak specifically to the need to manage preoperative opioid use in patients awaiting TJA, independent of any existing long-term opioid use.

Our findings for preoperative opioid use are in line with previous studies demonstrating a negative relationship between opioid use before TJA and subsequent healthcare utilization (e.g. readmissions,^25–31^ emergency department visits,^26,27,30^ opioid use,^8,11,31^ opioid overdose^26,27^) and medical costs.^28,29,31^ Most of these studies, however, look at shorter-term outcomes (up to 1 year). Our study shows that these effects are long-lasting and persist even 5 years after the surgery. We have also identified a link between preoperative opioid use and higher likelihood of receiving social benefits. While this association has not been previously found for TJA, it is consistent with the association between preoperative opioid use and lower rates of return to work after surgery identified in previous studies.^13–16^

Our finding that peri- and post-operative opioid use is associated with higher opioid use long-term after the TJA confirms the findings of previous studies.^11,32,33^ We also found that perioperative opioid use was associated with reduced days in hospital, hospitalization costs, and risk of death; we suggest caution in interpretation as this has not been confirmed in the literature. Although there are few studies on the broader health and economic effects of opioid use during surgical stay, one study found that opioid use within 3 days after a TJA is associated with increased risk of readmissions within 90 days.^34^ Post-discharge opioid prescribing was associated with increased long-term opioid and non-opioid prescribing and higher rate of injuries, but no other negative impacts were identified.

### Limitations

Our findings should be considered in light of several limitations of the study. First, our analysis only covers publicly funded surgeries, and the results may not be generalizable to privately funded surgeries. As access to publicly funded surgeries in NZ is rationed, the individuals in our analysis may generally be at a later stage of disease progression, be experiencing higher pain levels, and have a longer history of opioid use before surgery compared to individuals undergoing privately funded surgeries. Privately insured patients may have better access to postoperative care and enjoy better postoperative outcomes, although some evidence also suggests they have higher rates of peri- and postoperative opioid use.^35^ Second, our data only includes prescription drugs and does not cover the use of over-the-counter opioids (such as codeine with paracetamol). Assuming that over-the-counter opioids were used as a substitute for prescription opioids (i.e. that the proportion using opioids purchased over-the-counter is higher in patients not receiving prescription opioids), our results will underestimate the true association between opioid use and outcomes. Third, our findings for the trajectories of postoperative opioid use suggest that some patients may have relied on existing stocks of opioids rather than new dispensing in the first moth post-surgery; our post-discharge opioid use measure does not capture this use, which may bias our estimates for this period. Fourth, some of the outcomes (particularly the incidence of adverse events and health conditions) have low prevalence rates and therefore the results for these outcomes should be treated with caution. Finally, while we were able to control for an extensive set of measured baseline confounders, as this was an observational study we cannot rule out the possibility of confounding by unmeasured characteristics.

## Conclusion

This study indicates potential substantial health, economic, and social impacts of preoperative opioid use in patients with knee or hip osteoarthritis in primary care, who later undergo TJA. Despite higher numbers of TJA patients dispensed opioid medications in the peri- and post-operative periods following TJA, compared with the pre-operative period, our results find no compelling evidence that peri- and post-operative opioid prescription after TJA in NZ is associated with significant long-term detriments to healthcare utilization and healthcare costs, socio-economic outcomes, or incidence of adverse events/health conditions, at current rates of opioid dispensing.

These results highlight the importance of efforts to minimize preoperative opioid use, and suggest these should be a higher priority than addressing peri- and post-operative opioid prescription after TJA. Although opioid prescribing guidelines are available,^36–38^ there are challenges to reducing opioid prescribing in primary care^39–41^ and currently little evidence in the published literature to guide clinicians in how to address preoperative opioid use in people undergoing TJA^12^; this should be a priority for further research.

## Supplementary Material

cmad112_suppl_Supplementary_Appendix

## Data Availability

Access to the anonymised data used in this study was provided by Statistics NZ under the security and confidentiality provisions of the Statistics Act 1975. The data used in this study are not publicly available due to the strict security provisions of the IDI. Access to the IDI may be made available by Statistics NZ to approved researchers.

## IDI disclaimer

These results are not official statistics. They have been created for research purposes from the Integrated Data Infrastructure (IDI) which is carefully managed by Stats NZ. For more information about the IDI please visit https://www.stats.govt.nz/integrated-data/. The results are based in part on tax data supplied by Inland Revenue to Stats NZ under the Tax Administration Act 1994 for statistical purposes. Any discussion of data limitations or weaknesses is in the context of using the IDI for statistical purposes, and is not related to the data’s ability to support Inland Revenue’s core operational requirements.
