# Supplementary material for: The long-term impacts of opioid use before and after joint arthroplasty: matched cohort analysis of New Zealand linked register data
Source: Fam Pract. 2023 Dec 5;41(6):916–24. doi: 10.1093/fampra/cmad112 (PMC11636625; doi:10.1093/fampra/cmad112)
Supplement: cmad112_suppl_Supplementary_Appendix [file cmad112_suppl_supplementary_appendix.pdf]

The long-term impacts of opioid use before and after joint arthroplasty: matched cohort analysis of New Zealand linked register data

Appendix

Table A.1 – Outcomes used in the analysis

Variable	Dataset
Measured during the whole 5-year period	
Healthcare utilisation and healthcare costs	
Number of hospital admissions	MOH, Hospital Discharges
Number of days spent in hospital	MOH, Hospital Discharges
Number of emergency department visits	MOH, National Non-Admitted Patient Collection
Number of prescriptions dispensed	MOH, Pharmaceutical Collection
Number of opioid prescriptions dispensed	MOH, Pharmaceutical Collection
Opioid use in mg of morphine-equivalent daily dose	MOH, Pharmaceutical Collection
Outpatient and emergency department costs, NZD	MOH, National Non-Admitted Patient Collection
Laboratory testing costs, NZD	MOH, The Laboratory Claims Collection
Pharmaceutical costs, NZD	MOH, Pharmaceutical Collection
Hospitalization costs, NZD	MOH, Hospital Discharges
Total public healthcare costs, NZD (sum of outpatient, laboratory, pharmaceutical, hospitalization, and GMS costs)	Combination of MOH datasets
Accident and injury compensation, NZD	ACC Injury Claims
Socio-economic outcomes	
Total income, NZD	Inland Revenue
Income from wages and salaries, NZD	Inland Revenue
Number of months in employment (where employment defined as receiving wages/salaries)	Inland Revenue
Received social benefits (0, 1)	Inland Revenue
Number of months receiving social benefits	Inland Revenue
Was convicted (0, 1)	MOJ, Court Charges
Incidence of adverse events/health conditions	
Revision surgery (TJA on same joint) (0, 1)	MOH, Hospital Discharges
Death (0, 1)	Statistics NZ Personal Detail
Road accident (0, 1) or other injuries	ACC Injury Claims
Opioid overdose (0, 1) [1]	MOH, Hospital Discharges
Constipation (0, 1) [2]	MOH, Hospital Discharges
Bowel obstruction (0, 1) [3]	MOH, Hospital Discharges
Fracture (manual extraction of "fracture" codes from ICD-10) (0, 1)	MOH, Hospital Discharges
Fall (ICD-10 codes W01) (0, 1)	MOH, Hospital Discharges
Myocardial infarction (0, 1) [4]	MOH, Hospital Discharges
Heart failure (0,1) [4]	MOH, Hospital Discharges
Measured during each month of the 5-year period	
Healthcare utilisation and healthcare costs	
Was hospitalized (0, 1)	MOH, Hospital Discharges
Number of days spent in hospital	MOH, Hospital Discharges
Was at the emergency department (0, 1)	MOH, National Non-Admitted Patient Collection
Was dispensed medications (0, 1)	MOH, Pharmaceutical Collection
Was dispensed opioids (0, 1)	MOH, Pharmaceutical Collection
Total public healthcare costs	Combination of MOH datasets
Received accident and injury compensation (0, 1)	ACC Injury Claims
Socio-economic outcomes	
Total income, NZD	Inland Revenue
Income from wages and salaries, NZD	Inland Revenue
Was employed (0, 1)	Inland Revenue
Received social benefits (0, 1)	Inland Revenue

Abbreviations: MOH = Ministry of Health, GMS = General Medical Subsidies, ACC = Accident Compensation Corporation, MOJ = Ministry of Justice

Table A.2 – Mean values of the outcomes

Variable	Preoperative use		Perioperative use		Post-discharge use	
	Treatment	Control	Treatment	Control	Treatment	Control
**Healthcare utilization and healthcare costs**						
Hospitalizations	4.13	3.92	2.56	2.68	2.97	2.92
Days in hospital	18.48	16.62	7.90	9.47	10.15	11.42
Emergency department visits	2.89	2.71	2.20	2.25	2.54	2.45
Prescriptions dispensed	313.9	258.4	222.9	224.0	290.2	261.49
Opioid prescriptions (number)	30.69	10.87	16.14	12.97	24.21	10.50
Opioid prescriptions (OMEDD)	8.69	2.72	4.59	4.07	7.08	2.70
Healthcare costs (total)	56355	51380	26694	29814	31416	33981
Outpatient (NNPAC) costs	7036	6216	5906	6078	6350	6627
Lab costs	77.63	67.28	63.57	58.36	74.09	75.51
Pharmaceutical costs	4442	3771	3418	3582	4111	4092
Hospitalization costs	44791	41317	17300	20089	20872	23178
Accident and injury costs	3733	3590	2925	2349	3067	2688
**Socio-economic outcomes**						
Income (total)	107994	105925	123605	118748	111511	111784
Income (wage and salary)	40717	35903	56305	51822	42207	45547
Months on benefits	4.53	3.98	3.89	4.57	4.65	4.11
Months of employment	11.10	10.28	13.65	12.89	10.68	11.17
Received benefits	0.15	0.12	0.13	0.14	0.14	0.13
Convicted	0.01	0.01	0.01	0.02	0.02	0.01
**Incidence of adverse events/health conditions**						
TJA revision	0.04	0.03	0.03	0.03	0.03	0.02
Deceased	0.18	0.16	0.10	0.13	0.16	0.15
Road accident	0.03	0.03	0.03	0.03	0.04	0.03
Other injuries	2.44	2.36	2.14	2.09	2.36	2.18
Overdose	0.006	0.001	0.003	0.002	0.11	0.11
Constipation	0.17	0.17	0.08	0.09	0.02	0.01
Bowel obstruction	0.02	0.02	0.01	0.01	0.09	0.11
Fracture	0.19	0.20	0.07	0.09	0.09	0.10
Falls	0.14	0.13	0.07	0.08	0.05	0.05
Myocardial infarction	0.06	0.06	0.04	0.04	0.08	0.08
Heart failure	0.10	0.10	0.06	0.06	0.11	0.11

Abbreviations: OMEDD = Oral morphine equivalent daily dose, NNPAC = National Non-Admitted Patient Collection, TJA = total joint arthroplasty.

Table A.3 – Covariates used in matching

Variable	Description	Dataset
**Demographic characteristics**		
Age, years	Age at the time of surgery	Statistics NZ Personal Detail
Female	Female sex	Statistics NZ Personal Detail
Māori	Whether identifies as Māori	Statistics NZ Personal Detail
Pacific	Whether identifies as Pacific	Statistics NZ Personal Detail
**Geography** *(at the time of surgery)*		
Urban	Whether resides in an urban area	Statistics NZ Address Notification
**Prior health service use** *(measured over 9 months for preoperative use analysis and 12 months for perioperative and post-discharge use analyses, unless specified)*		
Number of hospitalizations	5 categories: 0, 1, 2, 3, >3	MOH, Hospital Discharges
Number of pharmaceutical prescriptions	11 categories: 0, non-0 values divided at deciles	MOH, Pharmaceutical Collection
Number of NSAISD medications	3 categories: 0, non-0 values divided at median	MOH, Pharmaceutical Collection
Number of non-opioid analgesics	3 categories: 0, non-0 values divided at median	MOH, Pharmaceutical Collection
Number of sedatives and hypnotics	3 categories: 0, non-0 values divided at median	MOH, Pharmaceutical Collection
Number of corticosteroids	3 categories: 0, non-0 values divided at median	MOH, Pharmaceutical Collection
Number of muscle relaxants	2 categories: 0, >0	MOH, Pharmaceutical Collection
Opioid use (in oral Morphine Equivalent Daily Dose);	5 categories: 0, non-0 values divided at quartiles	MOH, Pharmaceutical Collection
Number of opioids *during 3 months before surgery *,***	2 categories: 0, >0	MOH, Pharmaceutical Collection
Number of opioids *during hospital stay ***	2 categories: 0, >0	MOH, Pharmaceutical Collection
NNPAC costs	5 categories: divided at quintiles	MOH, National Non-Admitted Patient Collection
GMS costs	2 categories: 0, >0	MOH, General Medical Services
Lab costs	5 categories: 0, non-0 values divided at quartiles	MOH, The Laboratory Claims Collection
Pharmaceutical costs	5 categories: divided at quintiles	MOH, Pharmaceutical Collection
Hospitalization costs	5 categories: 0, non-0 values divided at quartiles	MOH, Hospital Discharges
Accident and injury costs	5 categories: 0, non-0 values divided at quartiles	ACC Injury Claims
Emergency department visits	4 categories: 0, 1, 2, >2	MOH, National Non-Admitted Patient Collection
**Socio-economic variables** *(measured over 9 months for preoperative use analysis and 12 months for perioperative and post-discharge use analyses, unless specified)*		
Income, 1000 NZD	Total income (including social benefits);<br>11 categories: 0, non-0 values divided at deciles	Inland Revenue
Employed	Was employed for at least 1 month	Inland Revenue
Benefits	Received social benefits for at least 1 month	Inland Revenue
Education level *(as of 2013 Census)*	4 dummy variables (base = No qualification)	2013 Census
- Secondary school		
- Post-secondary education		
- Tertiary education		
- Unknown education		
Number of convictions	2 categories: 0, >0	MOJ, Court Charges

Accidents *(measured over 9 months for preoperative and 12 months for perioperative and post-discharge use analyses)*		
Number of road accidents	2 categories: 0, >0	ACC Injury Claims
Number of other injuries	4 categories: 0, 1, 2, >2	ACC Injury Claims
Surgery characteristics		
Knee	Whether it was a knee surgery (base = Hip)	MOH, Hospital Discharges
Length of stay (days) **	10 categories: divided at deciles	
Discharged to another health facility **		
Year of surgery	2 dummy variables (base = 2011)	
- 2012		
- 2013		
Comorbid health conditions *(measured over 9 months for preoperative use analysis and 12 months for perioperative and post-discharge use analyses)*		
Depression<br>Anxiety<br>Psychoses<br>Hypertension/IHD/angina<br>Coagulopathy<br>Hyperlipidemia<br>Antiplatelet agents<br>Arrhythmias<br>Congestive heart failure<br>Diabetes<br>Hypothyroidism<br>Gastric acid disorder<br>Liver disease<br>Osteoporosis/Paget<br>Gout<br>Migraine	Identified using a combination of Elixhauser, Charlson, and the RxRisk-V measures [4,5].<br>Each is a dummy variable where the value of 1 indicates that an individual was identified to have that comorbidity according to at least one of the three measures. Some of the comorbidities identified by these measures were excluded from the analysis due to low prevalence.	MOH, Hospital Discharges and Pharmaceutical Collection

Notes: * and ** indicate that the variable was used only in the analysis of perioperative opioid use (*) and post-discharge opioid use (**).

Abbreviations: NNPAC = National Non-Admitted Patient Collection, GMS = General Medical Services, MOH = Ministry of Health, ACC = Accident Compensation Corporation, MOJ = Ministry of Justice.

Table A.4 – Matching Balancing Statistics Summary

Statistic	Preoperative use analysis	Perioperative use analysis	Post-discharge use analysis
SDM for the propensity score	0.04	0.002	0.008
RV for the propensity score	1.05	1.01	1.03
>0.5 (bad)	0	0	0
> 0.1 and ≤ 0.5 (good)	0	0	0
≤ 0.1 (**very good**)	**100**	**100**	**100**
≤0.5 (bad)	0	0	0
> 0.5 and ≤ 0.8 (good)	0	0	0
> 0.8 and ≤ 1.25 (**very good**)	**97.8**	**98.5**	**99.4**
> 1.25 and ≤ 2 (good)	2.2	1.5	0.6
> 2 (bad)	0	0	0
≤0.5 (bad)	0	0	0
> 0.5 and ≤ 0.8 (good)	0	0	0
> 0.8 and ≤ 1.25 (**very good**)	**97.8**	**98.5**	**99.4**
> 1.25 and ≤ 2 (good)	2.2	1.5	0.6
> 2 (bad)	0	0	0
Observations:			
Matched treated	6702	8967	7812
Unmatched treated	**30**	**0**	**0**
Matched control	3003	4647	4359
Unmatched control	8928	5052	6495
Model specification:			
Variables in exact matching	Basic	Basic	Basic
Replacement	Yes	Yes	Yes
Caliper 0.25 SD	Yes	No	No

Notes: Squares and interactions of continuous covariates were also included in the balance statistics analysis.

The Basic set of exact matching variables are: Female, Māori, Knee, Surgery Year.

Abbreviations: SDM = standard difference of means; RV = ratio of variances; SD = standard deviations.

Table A.5 – Sensitivity to model specification (preoperative use analysis)

Outcome	Main analysis		All covariates		No covariates	
	Est.	(95% CI)	Est.	(95% CI)	Est.	(95% CI)
**Healthcare utilization and healthcare costs**						
Hospitalizations	0.24	(0.03 to 0.45)	0.25	(0.04 to 0.45)	0.20	(−0.02 to 0.43)
Days in hospital	2.41	(0.96 to 3.85)	2.25	(0.83 to 3.66)	1.85	(0.35 to 3.36)
Emergency department visits	0.24	(−0.01 to 0.49)	0.24	(−0.01 to 0.49)	0.18	(−0.10 to 0.46)
Prescriptions dispensed	50.94	(32.15 to 69.73)	52.18	(34.55 to 69.81)	55.30	(34.39 to 76.21)
Opioid prescriptions (number)	19.67	(16.85 to 22.48)	18.19	(15.61 to 20.77)	19.81	(16.86 to 22.76)
Opioid prescriptions (OMEDD)	5.75	(4.27 to 7.24)	5.34	(3.85 to 6.83)	5.96	(4.46 to 7.47)
Healthcare costs (total)	5 602	(2 652 to 8 552)	5 342	(2 348 to 8 336)	4 951	(1 836 to 8 066)
Outpatient (NNPAC) costs	917.6	(206.7 to 1 629)	740.0	(46.9 to 1 433)	816.7	(3.4 to 1 630.0)
Lab costs	12.61	(5.06 to 20.17)	13.58	(6.30 to 20.86)	10.32	(0.32 to 20.33)
Pharmaceutical costs	296.0	(−179.1 to 771.2)	516.7	(−51.9 to 1 085)	669.2	(98.6 to 1 240)
Hospitalization costs	4 194	(1 504 to 6 884)	4 071	(1 349 to 6 792)	3 454	(661 to 6 247)
Accident and injury costs	176.3	(−527.5 to 880.0)	94.64	(−618.1 to 807.4)	141.8	(−574.5 to 858.0)
**Socio-economic outcomes**						
Income (total)	−2 137	(−5 200 to 927)	−1 188	(−4 918 to 2 542)	2 020	(−2 741 to 6 782)
Income (wage and salary)	−402.8	(−3 205 to 2 399)	876.9	(−2 802 to 4 555)	4 797	(−76 to 9 670)
Months on benefits	0.34	(−0.19 to 0.88)	0.31	(−0.25 to 0.88)	0.56	(−0.37 to 1.48)
Months of employment	−0.33	(−1.07 to 0.41)	−0.08	(−0.87 to 0.72)	0.82	(−0.39 to 2.02)
Received benefits	0.02	(0.01 to 0.03)	0.02	(0.00 to 0.03)	0.03	(0.00 to 0.05)
Convicted	0.00	(0.00 to 0.01)	0.00	(0.00 to 0.01)	0.00	(0.00 to 0.01)
**Incidence of adverse events/health conditions**						
TJA revision	0.01	(0.00 to 0.02)	0.01	(0.00 to 0.02)	0.01	(0.00 to 0.02)
Deceased	0.03	(0.01 to 0.06)	0.03	(0.00 to 0.05)	0.02	(0.00 to 0.05)
Road accident	0.00	(−0.01 to 0.01)	0.00	(−0.01 to 0.01)	0.00	(−0.01 to 0.02)
Other injuries	0.15	(−0.03 to 0.33)	0.16	(−0.02 to 0.33)	0.08	(−0.13 to 0.28)
Overdose	0.005	(0.003 to 0.008)	0.005	(0.003 to 0.007)	0.005	(0.003 to 0.008)
Constipation	0.00	(−0.02 to 0.02)	0.00	(−0.02 to 0.02)	−0.01	(−0.03 to 0.02)
Bowel obstruction	0.00	(−0.01 to 0.01)	0.00	(−0.01 to 0.01)	0.00	(−0.01 to 0.01)
Fracture	0.00	(−0.02 to 0.03)	0.00	(−0.02 to 0.02)	−0.01	(−0.03 to 0.02)
Falls	0.01	(−0.01 to 0.04)	0.01	(−0.01 to 0.03)	0.01	(−0.02 to 0.03)
Myocardial infarction	0.00	(−0.01 to 0.02)	0.00	(−0.01 to 0.02)	0.00	(−0.02 to 0.01)
Heart failure	0.00	(−0.02 to 0.02)	0.00	(−0.02 to 0.02)	−0.01	(−0.03 to 0.01)

Notes: Each estimate represents the result from a separate regression. The regressions in the main specification are adjusted for baseline covariates (age, sex, ethnicity, education level, geography, comorbidities, year of surgery, and type of surgery) and the pre-treatment value of the outcome. The regressions with all covariates also include pre-treatment values of all the other outcomes.
Abbreviations: OMEDD = Oral morphine equivalent daily dose, NNPAC = National Non-Admitted Patient Collection, TJA = total joint arthroplasty.

Table A.6 – Sensitivity to model specification (perioperative use analysis)

Outcome	Main analysis		All covariates		No covariates	
	Est.	(95% CI)	Est.	(95% CI)	Est.	(95% CI)
Healthcare utilization and healthcare costs						
Hospitalizations	−0.10	(−0.27 to 0.08)	−0.09	(−0.25 to 0.08)	−0.12	(−0.30 to 0.05)
Days in hospital	−1.51	(−2.35 to −0.66)	−1.48	(−2.31 to −0.66)	−1.57	(−2.42 to −0.71)
Emergency department visits	−0.06	(−0.20 to 0.08)	−0.03	(−0.17 to 0.10)	−0.05	(−0.19 to 0.10)
Prescriptions dispensed	1.28	(−8.48 to 11.03)	1.94	(−7.63 to 11.52)	−1.09	(−12.90 to 10.72)
Opioid prescriptions (number)	3.27	(1.29 to 5.26)	3.38	(1.42 to 5.34)	3.17	(1.10 to 5.24)
Opioid prescriptions (OMEDD)	0.54	(−1.18 to 2.26)	0.61	(−1.02 to 2.24)	0.52	(−1.21 to 2.25)
Healthcare costs (total)	−3 109	(−5 489 to −730)	−2 994	(−5 340 to −648)	−3 120	(−5 535 to −704)
Outpatient (NNPAC) costs	55.06	(−602.3 to 712.4)	−58.41	(−733.9 to 617.0)	−172.0	(−848.7 to 504.7)
Lab costs	6.61	(1.71 to 11.51)	6.84	(2.04 to 11.65)	5.21	(−0.82 to 11.24)
Pharmaceutical costs	−217.3	(−609.0 to 174.5)	−103.7	(−558.1 to 350.7)	−163.9	(−651.7 to 323.9)
Hospitalization costs	−2 903	(−4 982 to −823)	−2 838	(−4 881 to −794)	−2 788	(−4 860 to −716)
Accident and injury costs	598.2	(121.8 to 1 075)	591.9	(106.2 to 1 077.6)	575.8	(102.5 to 1 049)
Socio-economic outcomes						
Income (total)	1 307	(−1 722 to 4 336)	3 824	(−84 to 7 733)	4 856	(−55 to 9 767)
Income (wage and salary)	−893.0	(−3 755 to 1 969)	2 121	(−1 837 to 6 079)	4 482	(−671 to 9 634)
Months on benefits	−0.19	(−0.55 to 0.18)	−0.30	(−0.69 to 0.08)	−0.68	(−1.25 to −0.11)
Months of employment	0.12	(−0.43 to 0.66)	0.23	(−0.36 to 0.83)	0.76	(−0.16 to 1.69)
Received benefits	0.00	(−0.01 to 0.01)	0.00	(−0.01 to 0.01)	−0.01	(−0.02 to 0.01)
Convicted	0.00	(−0.01 to 0.00)	0.00	(−0.01 to 0.00)	0.00	(−0.01 to 0.00)
Incidence of adverse events/health conditions						
TJA revision	0.00	(−0.01 to 0.01)	0.00	(−0.01 to 0.01)	0.00	(−0.01 to 0.01)
Deceased	−0.03	(−0.04 to −0.02)	−0.03	(−0.04 to −0.02)	−0.02	(−0.04 to −0.01)
Road accident	0.00	(−0.01 to 0.00)	0.00	(−0.01 to 0.00)	0.00	(−0.01 to 0.00)
Other injuries	0.07	(−0.02 to 0.17)	0.07	(−0.02 to 0.17)	0.04	(−0.05 to 0.14)
Overdose	0.001	(−0.001 to 0.003)	0.001	(−0.001 to 0.003)	0.001	(−0.001 to 0.003)
Constipation	−0.01	(−0.02 to 0.00)	−0.01	(−0.02 to 0.00)	−0.01	(−0.02 to 0.00)
Bowel obstruction	0.00	(−0.01 to 0.00)	0.00	(−0.01 to 0.00)	0.00	(−0.01 to 0.00)
Fracture	−0.02	(−0.03 to 0.00)	−0.02	(−0.03 to 0.00)	−0.02	(−0.03 to 0.00)
Falls	−0.01	(−0.02 to 0.00)	−0.01	(−0.02 to 0.00)	−0.01	(−0.02 to 0.00)
Myocardial infarction	0.00	(−0.01 to 0.00)	0.00	(−0.01 to 0.00)	0.00	(−0.01 to 0.00)
Heart failure	−0.01	(−0.01 to 0.00)	−0.01	(−0.01 to 0.00)	−0.01	(−0.02 to 0.00)

Notes: Each estimate represents the result from a separate regression. The regressions in the main specification are adjusted for baseline covariates (age, sex, ethnicity, education level, geography, comorbidities, year of surgery, and type of surgery) and the pre-treatment value of the outcome. The regressions with all covariates also include pre-treatment values of all the other outcomes.
Abbreviations: OMEDD = Oral morphine equivalent daily dose, NNPAC = National Non-Admitted Patient Collection, TJA = total joint arthroplasty.

Table A.7 – Sensitivity to model specification (post-discharge use analysis)

Outcome	Main analysis		All covariates		No covariates	
	Est.	(95% CI)	Est.	(95% CI)	Est.	(95% CI)
**Healthcare utilization and healthcare costs**						
Hospitalizations	0.04	(−0.18 to 0.27)	0.03	(−0.18 to 0.25)	0.05	(−0.20 to 0.30)
Days in hospital	−1.42	(−2.84 to −0.01)	−1.48	(−2.82 to −0.13)	−1.27	(−2.83 to 0.29)
Emergency department visits	0.06	(−0.17 to 0.28)	0.07	(−0.15 to 0.28)	0.09	(−0.15 to 0.34)
Prescriptions dispensed	22.05	(7.39 to 36.71)	22.26	(8.02 to 36.50)	28.60	(10.72 to 46.48)
Opioid prescriptions (number)	13.41	(11.02 to 15.79)	12.69	(10.47 to 14.91)	13.70	(11.31 to 16.10)
Opioid prescriptions (OMEDD)	4.22	(3.19 to 5.26)	4.09	(3.08 to 5.11)	4.38	(3.30 to 5.46)
Healthcare costs (total)	−2 932	(−6 133 to 270)	−3 114	(−6 190 to −38)	−2 577	(−5 999 to 844)
Outpatient (NNPAC) costs	−184.8	(−897.6 to 528.1)	−198.3	(−930.5 to 533.9)	−279.7	(−1 060 to 500.2)
Lab costs	3.19	(−4.04 to 10.42)	1.07	(−6.41 to 8.54)	−1.45	(−11.31 to 8.41)
Pharmaceutical costs	101.6	(−473.3 to 676.6)	−23.83	(−654.0 to 606.3)	17.89	(−664.1 to 699.8)
Hospitalization costs	−2 756	(−5 519 to 7)	−2 893	(−5 574 to −213)	−2 315	(−5 213 to 584)
Accident and injury costs	393.4	(−190.2 to 976.9)	343.9	(−244.5 to 932.2)	378.2	(−218.1 to 974.5)
**Socio-economic outcomes**						
Income (total)	202.3	(−2 611to 3 015)	833.6	(−2 734 to 4 401)	−315.5	(−4 880 to 4 249)
Income (wage and salary)	−958.4	(−3 538 to 1 621)	−2 218	(−5 863 to 1 428)	−3 356	(−8 211 to 1 498)
Months on benefits	0.21	(−0.22 to 0.64)	0.41	(−0.04 to 0.86)	0.54	(−0.12 to 1.20)
Months of employment	−0.29	(−0.81 to 0.23)	−0.16	(−0.72 to 0.41)	−0.49	(−1.40 to 0.41)
Received benefits	0.01	(0.00 to 0.02)	0.01	(0.00 to 0.02)	0.01	(0.00 to 0.03)
Convicted	0.00	(0.00 to 0.01)	0.00	(0.00 to 0.01)	0.00	(0.00 to 0.01)
**Incidence of adverse events/health conditions**						
TJA revision	0.00	(0.00 to 0.01)	0.00	(0.00 to 0.01)	0.00	(0.00 to 0.01)
Deceased	−0.01	(−0.02 to 0.01)	−0.01	(−0.02 to 0.01)	0.00	(−0.01 to 0.02)
Road accident	0.00	(0.00 to 0.01)	0.00	(0.00 to 0.01)	0.00	(0.00 to 0.01)
Other injuries	0.19	(0.06 to 0.31)	0.16	(0.03 to 0.28)	0.17	(0.04 to 0.31)
Overdose	0.001	(−0.002 to 0.003)	0.000	(−0.002 to 0.003)	0.001	(−0.002 to 0.003)
Constipation	−0.01	(−0.02 to 0.01)	−0.01	(−0.02 to 0.01)	−0.01	(−0.02 to 0.01)
Bowel obstruction	0.00	(0.00 to 0.01)	0.00	(0.00 to 0.01)	0.00	(0.00 to 0.01)
Fracture	−0.02	(−0.04 to −0.01)	−0.02	(−0.04 to −0.01)	−0.02	(−0.03 to 0.00)
Falls	−0.01	(−0.03 to 0.00)	−0.01	(−0.03 to 0.00)	−0.01	(−0.02 to 0.00)
Myocardial infarction	0.00	(−0.01 to 0.01)	0.00	(−0.01 to 0.01)	0.00	(−0.01 to 0.01)
Heart failure	0.00	(−0.02 to 0.01)	0.00	(−0.02 to 0.01)	0.00	(−0.02 to 0.01)

Notes: Each estimate represents the result from a separate regression. The regressions in the main specification are adjusted for baseline covariates (age, sex, ethnicity, education level, geography, comorbidities, year of surgery, and type of surgery) and the pre-treatment value of the outcome. The regressions with all covariates also include pre-treatment values of all the other outcomes.

Abbreviations: OMEDD = Oral morphine equivalent daily dose, NNPAC = National Non-Admitted Patient Collection, TJA = total joint arthroplasty.

Table A.8 – Poisson regression

Outcome	Preoperative use		Perioperative use		Post-discharge use	
	MD	(95% CI)	MD	(95% CI)	MD	(95% CI)
**Healthcare utilization and healthcare costs**						
Hospitalizations	0.31	(−0.16 to 0.55)	−0.03	(−0.39 to 0.27)	−0.44	(−1.12 to 0.01)
Days in hospital	3.54	(1.11 to 5.44)	−1.29	(−2.28 to 0.43)	−3.97	(−8.47 to −1.74)
Emergency department visits	0.25	(−0.17 to 0.60)	−0.05	(−0.25 to 0.21)	−0.37	(−0.99 to 0.15)
Prescriptions dispensed	65.84	(36.30 to 94.52)	2.44	(−5.38 to 19.55)	28.18	(−2.86 to 46.83)
Opioid prescriptions (number)	22.27	(18.31 to 26.67)	3.82	(1.01 to 6.45)	13.12	(7.59 to 14.45)
Opioid prescriptions (OMEDD)	7.76	(5.51 to 9.90)	0.14	(−2.11 to 2.46)	4.59	(2.77 to 6.04)
Healthcare costs (total)	7 991	(3 461 to 12 083)	−2 634	(−5 619 to 1 818)	−8 255	(−16 210 to −2 033)
Outpatient (NNPAC) costs	813.4	(−435.2 to 1 562)	184.4	(−577.9 to 1 091)	−1 059	(−3 432 to 276)
Lab costs	16.66	(−1.94 to 32.18)	3.74	(−2.32 to 753.13)	−25.79	(−50.05 to 8.35)
Pharmaceutical costs	479.7	(−80.1 to 1 167.7)	−92.56	(−1 242 to 747.6)	−343.8	(−2 191 to 433.1)
Hospitalization costs	6 734	(2 718 to 10 346)	−2 507	(−4 956 to 1 045)	−6 586	(−13 172 to −1 728)
Accident and injury costs	403.5	(−864.3 to 1 237)	780.7	(195.9 to 1 522)	230.0	(−1 133 to 991.7)
**Socio-economic outcomes**						
Income (total)	2 265	(−7 712 to 5 325)	−4 000	(−8 851 to 4 055)	1 234	(−7 690 to 7 398)
Income (wage and salary)	4 173	(−7 778 to 8 279)	−7 801	(−10 976 to 2 077)	157.7	(−8 782 to 8 879)
Months on benefits	0.40	(−0.67 to 1.09)	−0.01	(−0.81 to 0.51)	−0.68	(−1.50 to 0.74)
Months of employment	−0.55	(−2.56 to 0.40)	−0.07	(−0.85 to 1.03)	0.30	(−1.28 to 0.96)
**Incidence of adverse events/health conditions**						
Other injuries	0.02	(−0.39 to 0.39)	0.12	(−0.05 to 0.26)	0.00	(−0.36 to 0.30)

Notes: Each estimate represents the result from a separate regression. The regressions are adjusted for baseline covariates (age, sex, ethnicity, education level, geography, comorbidities, year of surgery, and type of surgery) and the pre-treatment value of the outcome.
Abbreviations: OMEDD = Oral morphine equivalent daily dose, NNPAC = National Non-Admitted Patient Collection.

Table A.9 – Logistic regression

Outcome	Preoperative use		Perioperative use		Post-discharge use	
	RD	(95% CI)	RD	(95% CI)	RD	(95% CI)
**Socio-economic outcomes**						
Received benefits	0.01	(−0.01 to 0.03)	0.01	(−0.01 to 0.02)	0.01	(−0.01 to 0.03)
Convicted	0.00	(−0.01 to 0.01)	0.00	(−0.01 to 0.01)	0.01	(0.00 to 0.01)
**Incidence of adverse events/health conditions**						
TJA revision	0.01	(0.00 to 0.02)	0.00	(−0.01 to 0.01)	0.00	(−0.01 to 0.02)
Deceased	0.05	(0.02 to 0.09)	−0.02	(−0.03 to 0.00)	0.01	(−0.04 to 0.03)
Road accident	0.00	(−0.02 to 0.02)	−0.01	(−0.02 to 0.01)	0.00	(−0.02 to 0.02)
Overdose	0.005	(0.003 to 0.010)	0.001	(−0.002 to 0.003)	0.000	(−0.007 to 0.004)
Constipation	0.02	(−0.04 to 0.05)	−0.01	(−0.02 to 0.02)	−0.03	(−0.06 to 0.00)
Bowel obstruction	0.00	(0.00 to 0.01)	0.00	(−0.01 to 0.01)	0.00	(−0.01 to 0.01)
Fracture	0.01	(−0.03 to 0.05)	−0.02	(−0.03 to 0.01)	−0.05	(−0.09 to 0.00)
Falls	0.01	(−0.02 to 0.06)	−0.01	(−0.02 to 0.01)	−0.04	(−0.07 to 0.00)
Myocardial infarction	0.00	(−0.03 to 0.03)	0.00	(−0.02 to 0.01)	−0.01	(−0.04 to 0.01)
Heart failure	−0.02	(−0.05 to 0.03)	0.00	(−0.02 to 0.01)	−0.01	(−0.05 to 0.01)

Notes: Each estimate represents the result from a separate regression. The regressions are adjusted for baseline covariates (age, sex, ethnicity, education level, geography, comorbidities, year of surgery, and type of surgery) and the pre-treatment value of the outcome.
Abbreviations: TJA = total joint arthroplasty.

Table A.10 – Alternative matching model (with a 0.25SD caliper) for post-discharge use analysis

Outcome	Main analysis		Alt. matching model	
	Est.	(95% CI)	Est.	(95% CI)
**Healthcare utilization and healthcare costs**				
Hospitalizations	0.04	(−0.18 to 0.27)	0.11	(−0.08 to 0.29)
Days in hospital	−1.42	(−2.84 to −0.01)	−1.03	(−2.15 to 0.09)
Emergency department visits	0.06	(−0.17 to 0.28)	0.10	(−0.09 to 0.29)
Prescriptions dispensed	22.05	(7.39 to 36.71)	22.63	(9.07 to 36.18)
Opioid prescriptions (number)	13.41	(11.02 to 15.79)	13.02	(10.67 to 15.38)
Opioid prescriptions (OMEDD)	4.22	(3.19 to 5.26)	4.04	(3.04 to 5.05)
Healthcare costs (total)	−2 932	(−6 133 to 270)	−2 251	(−5 012 to 511)
Outpatient (NNPAC) costs	−184.8	(−897.6 to 528.1)	−118.1	(−798.3 to 562.0)
Lab costs	3.19	(−4.04 to 10.42)	3.61	(−3.16 to 10.39)
Pharmaceutical costs	101.6	(−473.3 to 676.6)	83.71	(−496.7 to 664.2)
Hospitalization costs	−2 756	(−5 519 to 7)	−2 133	(−4 539 to 274)
Accident and injury costs	393.4	(−190.2 to 976.9)	459.0	(−119.3 to 1 037)
**Socio-economic outcomes**				
Income (total)	202.3	(−2 611 to 3 015)	273.0	(−2 511 to 3 058)
Income (wage and salary)	−958.4	(−3 538 to 1 621)	−969.9	(−3 537 to 1 597)
Months on benefits	0.21	(−0.22 to 0.64)	0.18	(−0.24 to 0.60)
Months of employment	−0.29	(−0.81 to 0.23)	−0.29	(−0.80 to 0.23)
Received benefits	0.01	(0.00 to 0.02)	0.01	(0.00 to 0.02)
Convicted	0.00	(0.00 to 0.01)	0.00	(0.00 to 0.01)
**Incidence of adverse events/health conditions**				
TJA revision	0.00	(0.00 to 0.01)	0.01	(0.00 to 0.01)
Deceased	−0.01	(−0.02 to 0.01)	−0.01	(−0.02 to 0.01)
Road accident	0.00	(0.00 to 0.01)	0.01	(0.00 to 0.01)
Other injuries	0.19	(0.06 to 0.31)	0.21	(0.10 to 0.32)
Overdose	0.001	(−0.002 to 0.003)	0.001	(−0.002 to 0.003)
Constipation	−0.01	(−0.02 to 0.01)	−0.01	(−0.02 to 0.01)
Bowel obstruction	0.00	(0.00 to 0.01)	0.00	(0.00 to 0.01)
Fracture	−0.02	(−0.04 to −0.01)	−0.02	(−0.03 to −0.01)
Falls	−0.01	(−0.03 to 0.00)	−0.01	(−0.02 to 0.00)
Myocardial infarction	0.00	(−0.01 to 0.01)	0.00	(−0.01 to 0.01)
Heart failure	0.00	(−0.02 to 0.01)	0.00	(−0.01 to 0.01)

Notes: Each estimate represents the result from a separate regression. The regressions are adjusted for baseline covariates (age, sex, ethnicity, education level, geography, comorbidities, year of surgery, and type of surgery) and the pre-treatment value of the outcome.

Abbreviations: OMEDD = Oral morphine equivalent daily dose, NNPAC = National Non-Admitted Patient Collection, TJA = total joint arthroplasty.

Table A.11 – Effect of preoperative opioid use on long-term outcomes by ethnicity

Outcome	Māori (n = 1380)		Non- Māori (n = 12024)		Statist. Diff.
	Est.	(95% CI)	Est.	(95% CI)	p-Val.
**Healthcare utilization and healthcare costs**					
Hospitalizations	−0.07	(−0.60 to 0.45)	0.27	(0.05 to 0.50)	0.24
Days in hospital	0.89	(−2.12 to 3.90)	2.46	(0.88 to 4.03)	0.36
Emergency department visits	0.13	(−0.79 to 1.04)	0.27	(0.01 to 0.54)	0.76
Prescriptions dispensed	39.06	(−1.21 to 79.33)	50.36	(30.21 to 70.52)	0.62
Opioid prescriptions (number)	15.10	(7.63 to 22.56)	19.99	(16.99 to 22.99)	0.23
Opioid prescriptions (OMEDD)	6.64	(3.01 to 10.26)	5.60	(4.00 to 7.20)	0.61
Healthcare costs (total)	5 281	(−2 368 to 12 929)	5 646	(2 461 to 8 830)	0.93
Outpatient (NNPAC) costs	3 845	(−1 640 to 9 331)	655.1	(138.7 to 1 171.4)	0.25
Lab costs	−7.38	(−25.26 to 10.50)	14.16	(6.36 to 21.96)	0.03
Pharmaceutical costs	1 075	(139 to 2 010)	206.2	(−298.8 to 711.2)	0.11
Hospitalization costs	585.6	(−3 964 to 5 136)	4 511	(1 553 to 7 470)	0.15
Accident and injury costs	−1 399	(−3 369 to 572)	294.5	(−468.6 to 1 058)	0.11
**Socio-economic outcomes**					
Income (total)	−4 406	(−14 029 to 5 216)	−2 072	(−5 239 to 1 095)	0.65
Income (wage and salary)	−3 809	(−11 815 to 4 197)	−151.6	(−3 070 to 2 767)	0.40
Months on benefits	0.29	(−1.76 to 2.34)	0.32	(−0.18 to 0.82)	0.98
Months of employment	−0.06	(−1.96 to 1.85)	−0.38	(−1.16 to 0.40)	0.76
Received benefits	−0.01	(−0.05 to 0.03)	0.02	(0.01 to 0.03)	0.19
Convicted	0.00	(−0.03 to 0.03)	0.00	(0.00 to 0.01)	0.85
**Incidence of adverse events/health conditions**					
TJA revision	−0.02	(−0.06 to 0.02)	0.01	(0.00 to 0.02)	0.16
Deceased	0.02	(−0.04 to 0.07)	0.04	(0.01 to 0.06)	0.52
Road accident	0.03	(0.01 to 0.05)	0.00	(−0.01 to 0.01)	0.02
Other injuries	−0.21	(−0.67 to 0.25)	0.19	(0.00 to 0.38)	0.11
Overdose	0.003	(−0.001 to 0.007)	0.005	(0.003 to 0.008)	0.35
Constipation	−0.07	(−0.13 to −0.01)	0.01	(−0.02 to 0.03)	0.02
Bowel obstruction	−0.01	(−0.02 to 0.00)	0.00	(−0.01 to 0.01)	0.26
Fracture	0.01	(−0.04 to 0.05)	0.00	(−0.02 to 0.03)	0.78
Falls	0.03	(0.00 to 0.06)	0.01	(−0.01 to 0.03)	0.28
Myocardial infarction	0.01	(−0.01 to 0.04)	0.00	(−0.01 to 0.02)	0.42
Heart failure	0.00	(−0.05 to 0.05)	0.00	(−0.02 to 0.02)	0.89

Notes: Columns (1) and (2) report regression results separately for Māori and non-Māori respectively. Column (3) reports p-values of the interaction between treatment status and ethnicity in a regression using the whole sample but with subgroup interactions. Each estimate represents the result from a separate regression. All regressions are adjusted for baseline covariates (age, sex, ethnicity, education level, geography, comorbidities, year of surgery, and type of surgery) and the pre-treatment value of the outcome.

Abbreviations: OMEDD = Oral morphine equivalent daily dose, NNPAC = National Non-Admitted Patient Collection, TJA = total joint arthroplasty.

Table A.12 – Effect of preoperative opioid use on long-term outcomes by sex

Outcome	Female (n = 8028)		Male (n = 5376)		Statist. Diff.
	Est.	(95% CI)	Est.	(95% CI)	p-Val.
**Healthcare utilization and healthcare costs**					
Hospitalizations	0.31	(0.06 to 0.57)	0.14	(−0.22 to 0.51)	0.45
Days in hospital	2.58	(0.76 to 4.40)	2.13	(−0.14 to 4.41)	0.76
Emergency department visits	0.33	(0.05 to 0.61)	0.07	(−0.40 to 0.53)	0.35
Prescriptions dispensed	75.35	(54.75 to 95.94)	13.51	(−19.87 to 46.89)	0.00
Opioid prescriptions (number)	21.82	(18.04 to 25.61)	16.64	(12.30 to 20.99)	0.08
Opioid prescriptions (OMEDD)	6.61	(5.07 to 8.15)	4.47	(1.47 to 7.46)	0.21
Healthcare costs (total)	6 499	(2 714 to 10 285)	4 750	(172 to 9 329)	0.56
Outpatient (NNPAC) costs	757.1	(121.2 to 1 393)	1 229	(−180 to 2 638)	0.55
Lab costs	13.33	(3.14 to 23.52)	10.76	(1.50 to 20.01)	0.71
Pharmaceutical costs	405.7	(−218.7 to 1 030)	162.2	(−581.3 to 905.6)	0.62
Hospitalization costs	5 029	(1 490 to 8 568)	3 409	(−579 to 7 396)	0.55
Accident and injury costs	262.4	(−362.4 to 887.3)	14.57	(−1 451 to 1 480)	0.76
**Socio-economic outcomes**					
Income (total)	−4 001	(−7 620 to −381)	350.4	(−4 918 to 5 619)	0.18
Income (wage and salary)	−2 249	(−5 518 to 1 020)	2 039	(−2 952 to 7 029)	0.16
Months on benefits	0.18	(−0.42 to 0.78)	0.52	(−0.36 to 1.40)	0.53
Months of employment	−0.89	(−1.85 to 0.08)	0.54	(−0.59 to 1.67)	0.06
Received benefits	0.01	(−0.01 to 0.02)	0.03	(0.01 to 0.04)	0.08
Convicted	0.00	(−0.01 to 0.00)	0.01	(0.00 to 0.02)	0.03
**Incidence of adverse events/health conditions**					
TJA revision	0.01	(0.00 to 0.02)	0.00	(−0.02 to 0.01)	0.09
Deceased	0.02	(−0.01 to 0.06)	0.04	(0.02 to 0.07)	0.34
Road accident	0.01	(0.00 to 0.02)	−0.01	(−0.03 to 0.01)	0.12
Other injuries	0.15	(−0.09 to 0.39)	0.15	(−0.10 to 0.39)	0.99
Overdose	0.006	(0.004 to 0.009)	0.004	(0.000 to 0.009)	0.46
Constipation	0.00	(−0.03 to 0.03)	0.00	(−0.03 to 0.03)	0.95
Bowel obstruction	0.00	(−0.01 to 0.01)	0.00	(−0.01 to 0.01)	0.99
Fracture	0.00	(−0.03 to 0.03)	0.01	(−0.02 to 0.04)	0.47
Falls	0.01	(−0.02 to 0.04)	0.02	(0.00 to 0.05)	0.53
Myocardial infarction	−0.01	(−0.03 to 0.02)	0.02	(0.00 to 0.03)	0.13
Heart failure	−0.02	(−0.05 to 0.01)	0.03	(0.01 to 0.05)	0.00

Notes: Columns (1) and (2) report regression results separately for females and males respectively. Column (3) reports p-values of the interaction between treatment status and sex in a regression using the whole sample but with subgroup interactions. Each estimate represents the result from a separate regression. All regressions are adjusted for baseline covariates (age, sex, ethnicity, education level, geography, comorbidities, year of surgery, and type of surgery) and the pre-treatment value of the outcome.

Abbreviations: OMEDD = Oral morphine equivalent daily dose, NNPAC = National Non-Admitted Patient Collection, TJA = total joint arthroplasty.

Table A.13 – Effect of preoperative opioid use on long-term outcomes by surgery type

Outcome	Knee (n = 4746)		Hip (n = 8658)		Statist. Diff.
	Est.	(95% CI)	Est.	(95% CI)	p-Val.
Healthcare utilization and healthcare costs					
Hospitalizations	0.33	(−0.06 to 0.71)	0.20	(−0.05 to 0.45)	0.57
Days in hospital	1.84	(−0.63 to 4.31)	2.56	(0.76 to 4.37)	0.64
Emergency department visits	0.40	(−0.06 to 0.85)	0.18	(−0.10 to 0.46)	0.42
Prescriptions dispensed	45.56	(21.88 to 69.24)	53.96	(27.84 to 80.08)	0.64
Opioid prescriptions (number)	15.58	(11.92 to 19.23)	22.13	(18.28 to 25.98)	0.02
Opioid prescriptions (OMEDD)	4.84	(1.40 to 8.29)	6.27	(5.13 to 7.41)	0.44
Healthcare costs (total)	3 505	(−653 to 7 663)	6 734	(2 755 to 10 712)	0.27
Outpatient (NNPAC) costs	1 220	(−288 to 2 729)	798.2	(0.9 to 1 596)	0.63
Lab costs	16.55	(7.13 to 25.96)	8.65	(−1.16 to 18.45)	0.26
Pharmaceutical costs	61.93	(−730.4 to 854.2)	388.3	(−180.6 to 957.2)	0.51
Hospitalization costs	1 758	(−1 668 to 5 185)	5 435	(1 705 to 9 164)	0.15
Accident and injury costs	656.3	(−242.5 to 1 555)	−101.0	(−1 057 to 854.9)	0.26
Socio-economic outcomes					
Income (total)	−774.0	(−5 548 to 4 000)	−3 102	(−6 944 to 741)	0.46
Income (wage and salary)	2 224	(−2 548 to 6 997)	−2 089	(−5 374 to 1 196)	0.15
Months on benefits	0.48	(−0.33 to 1.29)	0.24	(−0.44 to 0.92)	0.65
Months of employment	−0.29	(−1.30 to 0.72)	−0.36	(−1.33 to 0.62)	0.92
Received benefits	0.02	(0.01 to 0.03)	0.02	(0.00 to 0.03)	0.72
Convicted	0.01	(0.00 to 0.01)	0.00	(−0.01 to 0.01)	0.35
Incidence of adverse events/health conditions					
TJA revision	−0.01	(−0.03 to 0.01)	0.01	(0.01 to 0.02)	0.03
Deceased	0.04	(0.02 to 0.06)	0.03	(0.00 to 0.06)	0.56
Road accident	0.01	(−0.01 to 0.02)	0.00	(−0.01 to 0.02)	0.51
Other injuries	0.26	(0.03 to 0.50)	0.08	(−0.16 to 0.32)	0.28
Overdose	0.007	(0.002 to 0.012)	0.005	(0.003 to 0.007)	0.44
Constipation	0.00	(−0.03 to 0.03)	0.00	(−0.03 to 0.03)	0.93
Bowel obstruction	0.00	(−0.01 to 0.01)	0.00	(−0.01 to 0.01)	0.53
Fracture	0.01	(−0.02 to 0.04)	0.00	(−0.03 to 0.03)	0.60
Falls	0.03	(0.01 to 0.05)	0.01	(−0.02 to 0.04)	0.25
Myocardial infarction	0.02	(0.01 to 0.03)	−0.01	(−0.03 to 0.01)	0.05
Heart failure	0.00	(−0.03 to 0.02)	0.00	(−0.02 to 0.03)	0.82

Notes: Columns (1) and (2) report regression results separately for knee and hip surgeries respectively. Column (3) reports p-values of the interaction between treatment status and type of surgery in a regression using the whole sample but with subgroup interactions. Each estimate represents the result from a separate regression. All regressions are adjusted for baseline covariates (age, sex, ethnicity, education level, geography, comorbidities, year of surgery, and type of surgery) and the pre-treatment value of the outcome.
Abbreviations: OMEDD = Oral morphine equivalent daily dose, NNPAC = National Non-Admitted Patient Collection, TJA = total joint arthroplasty.

Table A.14 – Effect of perioperative opioid use on long-term outcomes by ethnicity

Outcome	Māori (n = 1836)		Non- Māori (n = 16098)		Statist. Diff.
	Est.	(95% CI)	Est.	(95% CI)	p-Val.
Healthcare utilization and healthcare costs					
Hospitalizations	0.04	(−0.40 to 0.47)	−0.11	(−0.29 to 0.08)	0.55
Days in hospital	−0.87	(−3.46 to 1.73)	−1.51	(−2.38 to −0.63)	0.65
Emergency department visits	−0.13	(−0.63 to 0.37)	−0.05	(−0.19 to 0.09)	0.75
Prescriptions dispensed	14.63	(−12.00 to 41.26)	0.38	(−9.88 to 10.64)	0.33
Opioid prescriptions (number)	8.20	(1.38 to 15.02)	2.75	(0.68 to 4.82)	0.13
Opioid prescriptions (OMEDD)	4.36	(0.65 to 8.07)	0.10	(−1.76 to 1.96)	0.04
Healthcare costs (total)	−173.9	(−10 230 to 9 882)	−3 147	(−5 297 to −997)	0.57
Outpatient (NNPAC) costs	2 392	(−2 401 to 7 186)	−177.7	(−720.9 to 365.4)	0.29
Lab costs	0.50	(−12.82 to 13.82)	7.24	(1.96 to 12.51)	0.36
Pharmaceutical costs	425.3	(−392.0 to 1 243)	−304.6	(−731.5 to 122.3)	0.12
Hospitalization costs	−3 599	(−11 483 to 4 285)	−2 577	(−4 421 to −734)	0.80
Accident and injury costs	515.7	(−825.7 to 1 857)	621.4	(108.0 to 1 135)	0.88
Socio-economic outcomes					
Income (total)	10 613	(891 to 20 335)	208.3	(−2 973 to 3 390)	0.04
Income (wage and salary)	7 499	(−2 022 to 17 020)	−1 849	(−4 843 to 1 145)	0.06
Months on benefits	−0.07	(−1.47 to 1.32)	−0.17	(−0.54 to 0.21)	0.90
Months of employment	1.26	(−0.61 to 3.12)	0.00	(−0.57 to 0.57)	0.20
Received benefits	0.04	(0.02 to 0.07)	0.00	(−0.01 to 0.01)	0.00
Convicted	−0.01	(−0.03 to 0.01)	0.00	(−0.01 to 0.00)	0.35
Incidence of adverse events/health conditions					
TJA revision	−0.01	(−0.04 to 0.02)	0.00	(−0.01 to 0.01)	0.46
Deceased	−0.04	(−0.08 to 0.00)	−0.03	(−0.04 to −0.01)	0.40
Road accident	−0.03	(−0.07 to 0.00)	0.00	(−0.01 to 0.01)	0.10
Other injuries	0.26	(0.01 to 0.52)	0.05	(−0.05 to 0.15)	0.12
Overdose	0.001	(−0.001 to 0.003)	0.001	(−0.001 to 0.003)	0.78
Constipation	0.01	(−0.02 to 0.04)	−0.01	(−0.02 to 0.00)	0.12
Bowel obstruction	−0.01	(−0.02 to 0.00)	0.00	(−0.01 to 0.00)	0.22
Fracture	−0.01	(−0.03 to 0.02)	−0.02	(−0.03 to 0.00)	0.49
Falls	0.01	(−0.01 to 0.03)	−0.01	(−0.02 to 0.00)	0.05
Myocardial infarction	−0.01	(−0.03 to 0.01)	0.00	(−0.01 to 0.01)	0.69
Heart failure	−0.01	(−0.04 to 0.01)	−0.01	(−0.01 to 0.00)	0.57

Notes: Columns (1) and (2) report regression results separately for Māori and non-Māori respectively. Column (3) reports p-values of the interaction between treatment status and ethnicity in a regression using the whole sample but with subgroup interactions. Each estimate represents the result from a separate regression. All regressions are adjusted for baseline covariates (age, sex, ethnicity, education level, geography, comorbidities, year of surgery, and type of surgery) and the pre-treatment value of the outcome.

Abbreviations: OMEDD = Oral morphine equivalent daily dose, NNPAC = National Non-Admitted Patient Collection, TJA = total joint arthroplasty.

Table A.15 – Effect of perioperative opioid use on long-term outcomes by sex

Outcome	Female (n = 9672)		Male (n = 8262)		Statist. Diff.
	Est.	(95% CI)	Est.	(95% CI)	p-Val.
**Healthcare utilization and healthcare costs**					
Hospitalizations	−0.11	(−0.36 to 0.13)	−0.09	(−0.34 to 0.15)	0.90
Days in hospital	−1.57	(−2.84 to −0.31)	−1.41	(−2.48 to −0.34)	0.84
Emergency department visits	−0.03	(−0.20 to 0.15)	−0.12	(−0.34 to 0.10)	0.50
Prescriptions dispensed	7.64	(−6.44 to 21.71)	−6.52	(−19.79 to 6.76)	0.15
Opioid prescriptions (number)	4.44	(1.43 to 7.46)	1.90	(−0.60 to 4.40)	0.20
Opioid prescriptions (OMEDD)	1.11	(−0.26 to 2.47)	−0.11	(−3.33 to 3.11)	0.50
Healthcare costs (total)	−3 440	(−6 874 to −7)	−2 852	(−6 005 to 301)	0.80
Outpatient (NNPAC) costs	302.6	(−467.5 to 1 073)	−194.6	(−1 278 to 889)	0.46
Lab costs	11.16	(4.38 to 17.94)	0.99	(−6.12 to 8.09)	0.04
Pharmaceutical costs	−66.90	(−549.6 to 415.8)	−542.1	(−1 139 to 54.4)	0.23
Hospitalization costs	−3 564	(−6 690 to −438)	−2 319	(−4 865 to 227)	0.55
Accident and injury costs	72.59	(−351.8 to 497.0)	1 210	(300 to 2 120)	0.03
**Socio-economic outcomes**					
Income (total)	−460.9	(−4 371 to 3 449)	3 442	(−1 146 to 8 031)	0.21
Income (wage and salary)	−1 542	(−4 966 to 1 883)	63.74	(−4 461 to 4 588)	0.58
Months on benefits	−0.17	(−0.67 to 0.34)	−0.23	(−0.74 to 0.28)	0.88
Months of employment	0.18	(−0.50 to 0.87)	0.03	(−0.82 to 0.88)	0.78
Received benefits	0.01	(0.00 to 0.02)	0.00	(−0.02 to 0.01)	0.17
Convicted	0.00	(0.00 to 0.00)	0.00	(−0.01 to 0.01)	0.35
**Incidence of adverse events/health conditions**					
TJA revision	0.00	(−0.01 to 0.01)	0.00	(−0.01 to 0.01)	0.46
Deceased	−0.02	(−0.04 to −0.01)	−0.03	(−0.05 to −0.02)	0.38
Road accident	0.00	(−0.01 to 0.01)	−0.01	(−0.02 to 0.00)	0.16
Other injuries	0.04	(−0.09 to 0.17)	0.12	(−0.01 to 0.25)	0.36
Overdose	0.000	(−0.003 to 0.003)	0.002	(−0.001 to 0.004)	0.37
Constipation	−0.01	(−0.02 to 0.01)	−0.01	(−0.03 to 0.00)	0.42
Bowel obstruction	0.00	(0.00 to 0.01)	−0.01	(−0.01 to 0.00)	0.16
Fracture	−0.02	(−0.04 to 0.00)	−0.01	(−0.03 to 0.00)	0.42
Falls	−0.02	(−0.03 to −0.01)	0.00	(−0.01 to 0.01)	0.04
Myocardial infarction	0.00	(−0.01 to 0.01)	−0.01	(−0.02 to 0.01)	0.55
Heart failure	−0.01	(−0.02 to 0.00)	0.00	(−0.01 to 0.01)	0.25

Notes: Columns (1) and (2) report regression results separately for females and males respectively. Column (3) reports p-values of the interaction between treatment status and sex in a regression using the whole sample but with subgroup interactions. Each estimate represents the result from a separate regression. All regressions are adjusted for baseline covariates (age, sex, ethnicity, education level, geography, comorbidities, year of surgery, and type of surgery) and the pre-treatment value of the outcome.
Abbreviations: OMEDD = Oral morphine equivalent daily dose, NNPAC = National Non-Admitted Patient Collection, TJA = total joint arthroplasty.

Table A.16 – Effect of perioperative opioid use on long-term outcomes by surgery type

Outcome	Knee (n = 8892)		Hip (n = 9042)		Statist. Diff.
	Est.	(95% CI)	Est.	(95% CI)	p-Val.
**Healthcare utilization and healthcare costs**					
Hospitalizations	0.04	(−0.18 to 0.26)	−0.25	(−0.51 to 0.02)	0.11
Days in hospital	−1.73	(−3.03 to −0.43)	−1.40	(−2.50 to −0.31)	0.71
Emergency department visits	−0.03	(−0.25 to 0.19)	−0.08	(−0.25 to 0.09)	0.70
Prescriptions dispensed	−3.22	(−16.31 to 9.86)	4.72	(−9.63 to 19.07)	0.42
Opioid prescriptions (number)	−0.43	(−2.68 to 1.82)	6.89	(3.68 to 10.10)	0.00
Opioid prescriptions (OMEDD)	−0.61	(−3.84 to 2.61)	1.80	(0.72 to 2.88)	0.16
Healthcare costs (total)	−2 498	(−5 903 to 908)	−3 609	(−6 538 to −681)	0.63
Outpatient (NNPAC) costs	414.7	(−762.3 to 1 592)	−230.2	(−874.1 to 413.8)	0.34
Lab costs	6.97	(−1.11 to 15.04)	6.53	(0.87 to 12.19)	0.93
Pharmaceutical costs	−495.5	(−1 156 to 165.1)	51.23	(−390.4 to 493.0)	0.18
Hospitalization costs	−2 364	(−5 239 to 511)	−3 531	(−6 170 to −891)	0.56
Accident and injury costs	1 094	(306 to 1 882)	134.7	(−401.2 to 670.5)	0.05
**Socio-economic outcomes**					
Income (total)	1 130	(−3 345 to 5 605)	1 497	(−2 521 to 5 516)	0.91
Income (wage and salary)	−548.8	(−4 858 to 3 760)	−1 357	(−5 136 to 2 422)	0.78
Months on benefits	−0.24	(−0.78 to 0.31)	−0.13	(−0.62 to 0.35)	0.78
Months of employment	0.48	(−0.32 to 1.28)	−0.28	(−1.01 to 0.46)	0.17
Received benefits	0.00	(−0.01 to 0.01)	0.00	(−0.01 to 0.02)	0.74
Convicted	0.00	(−0.01 to 0.01)	0.00	(−0.01 to 0.00)	0.28
**Incidence of adverse events/health conditions**					
TJA revision	0.00	(−0.01 to 0.01)	0.00	(−0.01 to 0.01)	0.26
Deceased	−0.02	(−0.04 to 0.00)	−0.03	(−0.05 to −0.02)	0.20
Road accident	−0.01	(−0.02 to 0.00)	0.00	(−0.01 to 0.01)	0.32
Other injuries	0.12	(−0.01 to 0.26)	0.02	(−0.10 to 0.15)	0.29
Overdose	0.000	(−0.003 to 0.003)	0.001	(−0.001 to 0.003)	0.61
Constipation	−0.01	(−0.03 to 0.01)	−0.01	(−0.02 to 0.00)	0.96
Bowel obstruction	0.00	(0.00 to 0.01)	−0.01	(−0.01 to 0.00)	0.02
Fracture	−0.01	(−0.02 to 0.01)	−0.02	(−0.04 to −0.01)	0.24
Falls	0.00	(−0.01 to 0.01)	−0.02	(−0.03 to −0.01)	0.04
Myocardial infarction	0.00	(−0.01 to 0.01)	0.00	(−0.01 to 0.01)	0.84
Heart failure	−0.01	(−0.02 to 0.01)	−0.01	(−0.02 to 0.00)	0.80

Notes: Columns (1) and (2) report regression results separately for knee and hip surgeries respectively. Column (3) reports p-values of the interaction between treatment status and type of surgery in a regression using the whole sample but with subgroup interactions. Each estimate represents the result from a separate regression. All regressions are adjusted for baseline covariates (age, sex, ethnicity, education level, geography, comorbidities, year of surgery, and type of surgery) and the pre-treatment value of the outcome.
Abbreviations: OMEDD = Oral morphine equivalent daily dose, NNPAC = National Non-Admitted Patient Collection, TJA = total joint arthroplasty.

Table A.17 – Effect of post-discharge opioid use on long-term outcomes by ethnicity

Outcome	Māori (n = 1668)		Non- Māori (n = 13956)		Statist. Diff.
	Est.	(95% CI)	Est.	(95% CI)	p-Val.
Healthcare utilization and healthcare costs					
Hospitalizations	−0.46	(−0.99 to 0.06)	0.11	(−0.13 to 0.36)	0.05
Days in hospital	−3.00	(−5.61 to −0.39)	−1.21	(−2.72 to 0.31)	0.24
Emergency department visits	0.06	(−0.57 to 0.68)	0.06	(−0.17 to 0.30)	0.98
Prescriptions dispensed	4.56	(−38.79 to 47.92)	24.01	(8.82 to 39.21)	0.41
Opioid prescriptions (number)	12.93	(3.74 to 22.12)	13.45	(11.00 to 15.90)	0.91
Opioid prescriptions (OMEDD)	5.54	(1.07 to 10.01)	4.04	(3.02 to 5.05)	0.52
Healthcare costs (total)	−3 049	(−9 613 to 3 516)	−2 805	(−6 262 to 652)	0.95
Outpatient (NNPAC) costs	−716.6	(−5 291 to 3 858)	−101.2	(−693.4 to 491.1)	0.79
Lab costs	−18.23	(−44.72 to 8.26)	5.77	(−1.41 to 12.94)	0.09
Pharmaceutical costs	355.2	(−1 013 to 1 724)	79.32	(−550.4 to 709.0)	0.72
Hospitalization costs	−3 290	(−6 600 to 19)	−2 724	(−5 752 to 304)	0.80
Accident and injury costs	−1 169	(−2 913 to 575)	565.1	(−59.5 to 1 190)	0.07
Socio-economic outcomes					
Income (total)	4 541	(−4 925 to 14 006)	−348.6	(−3 253 to 2 556)	0.33
Income (wage and salary)	2 325	(−6 990 to 11 640)	−1 366	(−4 010 to 1 278)	0.45
Months on benefits	2.17	(0.45 to 3.89)	−0.02	(−0.44 to 0.41)	0.01
Months of employment	−0.41	(−2.32 to 1.51)	−0.27	(−0.80 to 0.26)	0.89
Received benefits	0.02	(−0.02 to 0.05)	0.01	(0.00 to 0.02)	0.60
Convicted	0.02	(0.00 to 0.04)	0.00	(0.00 to 0.01)	0.19
Incidence of adverse events/health conditions					
TJA revision	0.02	(−0.01 to 0.04)	0.00	(0.00 to 0.01)	0.21
Deceased	−0.04	(−0.09 to 0.01)	0.00	(−0.02 to 0.01)	0.16
Road accident	−0.02	(−0.07 to 0.02)	0.01	(0.00 to 0.02)	0.16
Other injuries	0.12	(−0.15 to 0.38)	0.20	(0.06 to 0.33)	0.61
Overdose	−0.002	(−0.012 to 0.008)	0.001	(−0.002 to 0.004)	0.53
Constipation	−0.03	(−0.06 to 0.01)	−0.01	(−0.02 to 0.01)	0.27
Bowel obstruction	−0.01	(−0.03 to 0.00)	0.00	(0.00 to 0.01)	0.13
Fracture	−0.05	(−0.09 to −0.01)	−0.02	(−0.03 to 0.00)	0.19
Falls	−0.01	(−0.04 to 0.02)	−0.01	(−0.03 to 0.00)	0.85
Myocardial infarction	0.00	(−0.04 to 0.03)	0.00	(−0.01 to 0.01)	0.89
Heart failure	−0.05	(−0.10 to 0.00)	0.00	(−0.01 to 0.01)	0.04

Notes: Columns (1) and (2) report regression results separately for Māori and non-Māori respectively. Column (3) reports p-values of the interaction between treatment status and ethnicity in a regression using the whole sample but with subgroup interactions. Each estimate represents the result from a separate regression. All regressions are adjusted for baseline covariates (age, sex, ethnicity, education level, geography, comorbidities, year of surgery, and type of surgery) and the pre-treatment value of the outcome.
Abbreviations: OMEDD = Oral morphine equivalent daily dose, NNPAC = National Non-Admitted Patient Collection, TJA = total joint arthroplasty.

Table A.18 – Effect of post-discharge opioid use on long-term outcomes by sex

Outcome	Female (n = 8700)		Male (n = 6924)		Statist. Diff.
	Est.	(95% CI)	Est.	(95% CI)	p-Val.
**Healthcare utilization and healthcare costs**					
Hospitalizations	0.16	(−0.08 to 0.40)	−0.09	(−0.48 to 0.30)	0.28
Days in hospital	−0.43	(−1.93 to 1.06)	−2.59	(−4.96 to −0.22)	0.13
Emergency department visits	0.10	(−0.16 to 0.37)	0.00	(−0.36 to 0.37)	0.68
Prescriptions dispensed	35.83	(17.12 to 54.55)	4.94	(−17.32 to 27.21)	0.04
Opioid prescriptions (number)	14.82	(11.56 to 18.08)	11.46	(7.98 to 14.95)	0.17
Opioid prescriptions (OMEDD)	4.29	(2.92 to 5.66)	4.18	(2.56 to 5.81)	0.92
Healthcare costs (total)	−2 813	(−6 665 to 1 039)	−3 243	(−8 226 to 1 741)	0.89
Outpatient (NNPAC) costs	−55.78	(−758.6 to 647.1)	−327.0	(−1 644 to 990.0)	0.72
Lab costs	6.24	(−3.00 to 15.49)	−0.85	(−12.09 to 10.39)	0.34
Pharmaceutical costs	−216.9	(−1 086 to 651.9)	470.0	(−150.4 to 1 090)	0.21
Hospitalization costs	−2 439	(−5 897 to 1 019)	−3 351	(−7 493 to 791)	0.74
Accident and injury costs	527.1	(51.9 to 1 002)	257.2	(−910.8 to 1 425)	0.67
**Socio-economic outcomes**					
Income (total)	133.6	(−3 169 to 3 436)	53.26	(−4 667 to 4 774)	0.98
Income (wage and salary)	−1 085	(−4 014 to 1 843)	−1 092	(−5 514 to 3 330)	1.00
Months on benefits	0.16	(−0.43 to 0.75)	0.29	(−0.30 to 0.88)	0.77
Months of employment	−0.14	(−0.76 to 0.48)	−0.51	(−1.38 to 0.36)	0.50
Received benefits	0.01	(−0.01 to 0.02)	0.01	(0.00 to 0.03)	0.47
Convicted	0.00	(0.00 to 0.01)	0.01	(0.00 to 0.01)	0.61
**Incidence of adverse events/health conditions**					
TJA revision	0.01	(0.00 to 0.02)	0.00	(−0.01 to 0.01)	0.37
Deceased	−0.02	(−0.04 to 0.00)	0.00	(−0.02 to 0.03)	0.15
Road accident	0.00	(−0.01 to 0.01)	0.00	(−0.01 to 0.02)	0.92
Other injuries	0.29	(0.15 to 0.44)	0.05	(−0.14 to 0.24)	0.05
Overdose	0.001	(−0.003 to 0.005)	0.001	(−0.003 to 0.004)	0.97
Constipation	0.00	(−0.02 to 0.02)	−0.02	(−0.04 to 0.01)	0.32
Bowel obstruction	0.01	(0.00 to 0.01)	−0.01	(−0.02 to 0.00)	0.01
Fracture	−0.01	(−0.03 to 0.01)	−0.03	(−0.06 to −0.01)	0.16
Falls	0.00	(−0.02 to 0.01)	−0.02	(−0.04 to 0.00)	0.26
Myocardial infarction	0.00	(−0.01 to 0.01)	0.00	(−0.02 to 0.02)	0.91
Heart failure	0.00	(−0.01 to 0.01)	−0.01	(−0.03 to 0.02)	0.66

Notes: Columns (1) and (2) report regression results separately for females and males respectively. Column (3) reports p-values of the interaction between treatment status and sex in a regression using the whole sample but with subgroup interactions. Each estimate represents the result from a separate regression. All regressions are adjusted for baseline covariates (age, sex, ethnicity, education level, geography, comorbidities, year of surgery, and type of surgery) and the pre-treatment value of the outcome.

Abbreviations: OMEDD = Oral morphine equivalent daily dose, NNPAC = National Non-Admitted Patient Collection, TJA = total joint arthroplasty.

Table A.19 – Effect of post-discharge opioid use on long-term outcomes by surgery type

Outcome	Knee (n = 7998)		Hip (n = 7626)		Statist. Diff.
	Est.	(95% CI)	Est.	(95% CI)	p-Val.
**Healthcare utilization and healthcare costs**					
Hospitalizations	−0.11	(−0.48 to 0.27)	0.17	(−0.06 to 0.41)	0.22
Days in hospital	−2.24	(−4.59 to 0.11)	−0.61	(−2.05 to 0.82)	0.25
Emergency department visits	0.00	(−0.36 to 0.35)	0.11	(−0.16 to 0.37)	0.63
Prescriptions dispensed	−4.77	(−26.94 to 17.39)	48.54	(30.60 to 66.48)	0.00
Opioid prescriptions (number)	8.22	(6.27 to 10.16)	18.67	(14.35 to 22.99)	0.00
Opioid prescriptions (OMEDD)	3.30	(1.80 to 4.81)	5.09	(3.73 to 6.45)	0.08
Healthcare costs (total)	−4 981	(−10 203 to 240)	−812.7	(−4 489.5 to 2 864.2)	0.20
Outpatient (NNPAC) costs	−669.5	(−1 811.0 to 471.9)	305.7	(−477.6 to 1 088.9)	0.17
Lab costs	7.80	(−1.53 to 17.13)	−0.98	(−11.86 to 9.89)	0.23
Pharmaceutical costs	−489.8	(−1 520 to 540.4)	659.4	(135.1 to 1 183.6)	0.05
Hospitalization costs	−4 282	(−8 744 to 180)	−1 417	(−4 748 to 1 913)	0.31
Accident and injury costs	526.9	(−363.5 to 1 417)	283.2	(−442.4 to 1 008.8)	0.68
**Socio-economic outcomes**					
Income (total)	−491.3	(−4 797.5 to 3 815)	816.2	(−2 766 to 4 398)	0.65
Income (wage and salary)	−690.7	(−4 769 to 3 388)	−1 194	(−4 341 to 1 954)	0.85
Months on benefits	0.47	(−0.18 to 1.13)	−0.05	(−0.60 to 0.50)	0.23
Months of employment	−0.10	(−0.88 to 0.68)	−0.45	(−1.14 to 0.23)	0.50
Received benefits	0.01	(0.00 to 0.03)	0.00	(−0.01 to 0.01)	0.29
Convicted	0.00	(0.00 to 0.01)	0.00	(0.00 to 0.01)	0.73
**Incidence of adverse events/health conditions**					
TJA revision	0.00	(−0.01 to 0.01)	0.01	(0.00 to 0.01)	0.57
Deceased	−0.01	(−0.02 to 0.01)	0.00	(−0.02 to 0.02)	0.78
Road accident	0.00	(−0.01 to 0.02)	0.01	(0.00 to 0.02)	0.74
Other injuries	0.16	(−0.03 to 0.35)	0.21	(0.06 to 0.36)	0.68
Overdose	0.002	(−0.001 to 0.005)	−0.001	(−0.006 to 0.003)	0.18
Constipation	−0.02	(−0.04 to 0.00)	0.00	(−0.01 to 0.02)	0.10
Bowel obstruction	0.00	(−0.01 to 0.01)	0.00	(−0.01 to 0.01)	0.45
Fracture	−0.03	(−0.05 to −0.01)	−0.01	(−0.03 to 0.00)	0.37
Falls	−0.02	(−0.04 to 0.00)	−0.01	(−0.03 to 0.01)	0.67
Myocardial infarction	0.00	(−0.02 to 0.01)	0.00	(−0.02 to 0.01)	0.90
Heart failure	−0.01	(−0.03 to 0.01)	0.00	(−0.01 to 0.02)	0.29

Notes: Columns (1) and (2) report regression results separately for knee and hip surgeries respectively. Column (3) reports p-values of the interaction between treatment status and type of surgery in a regression using the whole sample but with subgroup interactions. Each estimate represents the result from a separate regression. All regressions are adjusted for baseline covariates (age, sex, ethnicity, education level, geography, comorbidities, year of surgery, and type of surgery) and the pre-treatment value of the outcome.
Abbreviations: OMEDD = Oral morphine equivalent daily dose, NNPAC = National Non-Admitted Patient Collection, TJA = total joint arthroplasty.

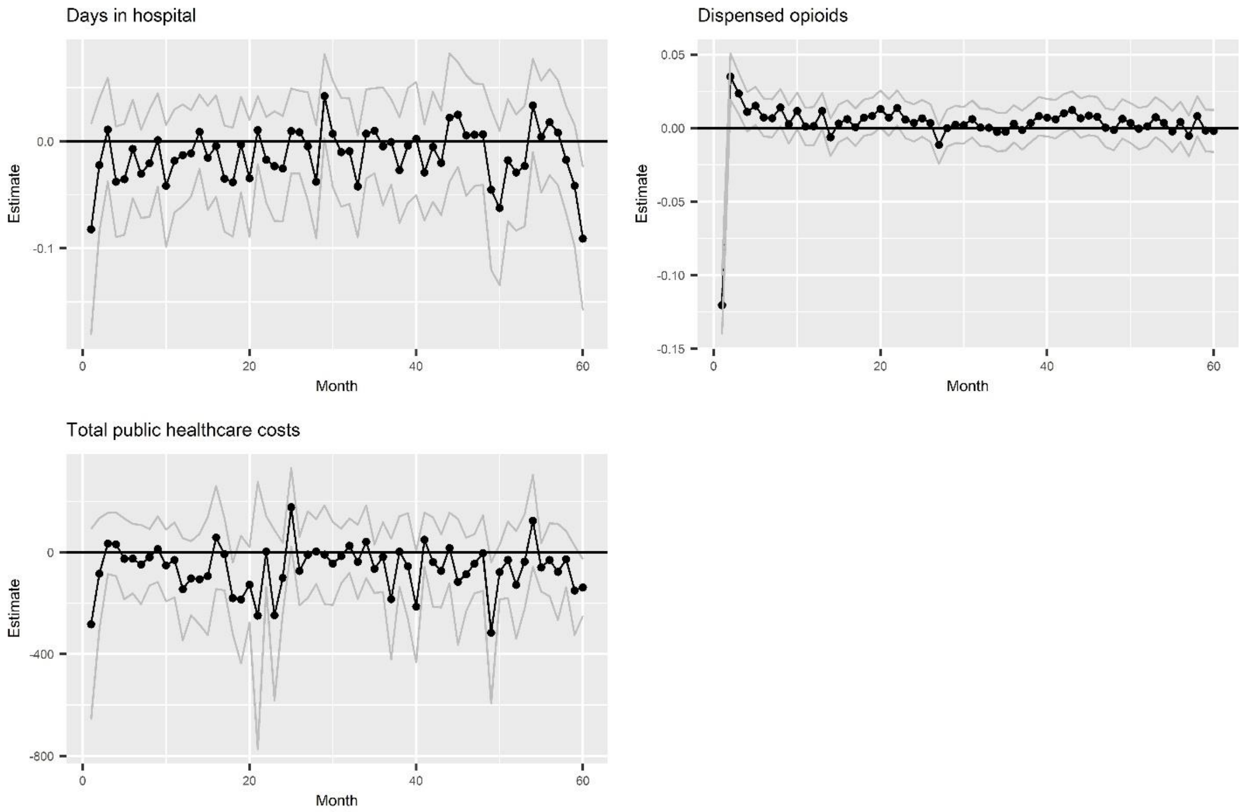

Figure A.1 – Trajectories of effect estimates for perioperative opioid use

Note: The grey lines represent the 95% confidence intervals.

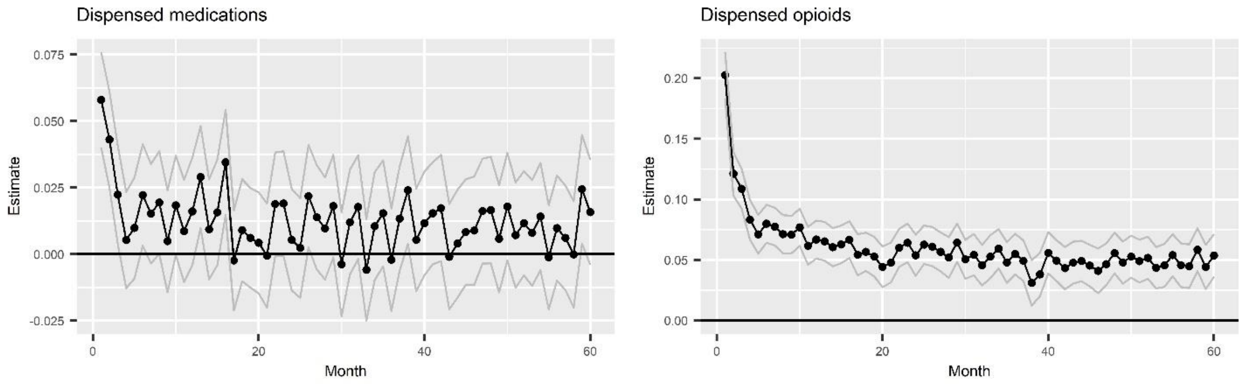

Figure A.2 – Trajectories of effect estimates for post-discharge opioid use

Note: The grey lines represent the 95% confidence intervals.

References

1 Green CA, Perrin NA, Janoff SL, Campbell CI, Chilcoat HD, Coplan PM. Assessing the accuracy of opioid overdose and poisoning codes in diagnostic information from electronic health records, claims data, and death records. *Pharmacoepidemiol. Drug Saf.* 2017;26(5):509-517.

2 Tonelli M, Wiebe N, Fortin M, et al. Methods for identifying 30 chronic conditions: application to administrative data. BMC Medical Inform. Decis. Mak. 2016;15(31).

3 Gandle C, Scott FI, Waljee A, Vajravelu RK, Sansgiry S, Hou JK. Development and Validation of an Administrative Codes Algorithm to Identify Abdominal Surgery and Bowel Obstruction in Patients With Inflammatory Bowel Disease. Crohn's & Colitis 360 2021;3(1):otab010.

4 Quan H, Sundararajan V, Halfon P, et al. Coding Algorithms for Defining Comorbidities in ICD-9-CM and ICD-10 Administrative Data. Med. Care. 2005; 43(11): 1130-9.

5 Sloan KL, Sales AE, Liu CF, Fishman P, Nichol P, Suzuki NT, et al. Construction and characteristics of the RxRisk-V: a VA-adapted pharmacy-based case-mix instrument. Med Care 2003;41(6):761–74.

6 **The RECORD statement – checklist of items, extended from the STROBE statement, that should be reported in observational studies using routinely collected health data.**

	Item No.	STROBE items	Location in manuscript where items are reported	RECORD items	Location in manuscript where items are reported
**Title and abstract**					
	1	(a) Indicate the study's design with a commonly used term in the title or the abstract (b) Provide in the abstract an informative and balanced summary of what was done and what was found	p.1, 2	RECORD 1.1: The type of data used should be specified in the title or abstract. When possible, the name of the databases used should be included.<br><br>RECORD 1.2: If applicable, the geographic region and timeframe within which the study took place should be reported in the title or abstract.<br><br>RECORD 1.3: If linkage between databases was conducted for the study, this should be clearly stated in the title or abstract.	p.1<br><br>pp.1, 2
**Introduction**					
Background rationale	2	Explain the scientific background and rationale for the investigation being reported	p.3		
Objectives	3	State specific objectives, including any prespecified hypotheses	p.3		
**Methods**					
Study Design	4	Present key elements of study design early in the paper	p.2		
Setting	5	Describe the setting, locations, and relevant dates, including periods of recruitment, exposure,	n/a		

		follow-up, and data collection			
Participants	6	*(a) Cohort study* - Give the eligibility criteria, and the sources and methods of selection of participants. Describe methods of follow-up<br><br>*Case-control study* - Give the eligibility criteria, and the sources and methods of case ascertainment and control selection. Give the rationale for the choice of cases and controls<br><br>*Cross-sectional study* - Give the eligibility criteria, and the sources and methods of selection of participants<br><br>*(b) Cohort study* - For matched studies, give matching criteria and number of exposed and unexposed<br><br>*Case-control study* - For matched studies, give matching criteria and the number of controls per case		RECORD 6.1: The methods of study population selection (such as codes or algorithms used to identify subjects) should be listed in detail. If this is not possible, an explanation should be provided.<br><br>RECORD 6.2: Any validation studies of the codes or algorithms used to select the population should be referenced. If validation was conducted for this study and not published elsewhere, detailed methods and results should be provided.<br><br>RECORD 6.3: If the study involved linkage of databases, consider use of a flow diagram or other graphical display to demonstrate the data linkage process, including the number of individuals with linked data at each stage.	p.4
Variables	7	Clearly define all outcomes, exposures, predictors, potential confounders, and effect modifiers. Give diagnostic criteria, if applicable.		RECORD 7.1: A complete list of codes and algorithms used to classify exposures, outcomes, confounders, and effect modifiers should be provided. If these cannot be reported, an explanation should be provided.	p.4,19-21<br><br>(ref. 1-5 in Appendix)
Data sources/ measurement	8	For each variable of interest, give sources of data and details of methods of assessment (measurement).	pp.19-21		

		Describe comparability of assessment methods if there is more than one group			
Bias	9	Describe any efforts to address potential sources of bias	p.5		
Study size	10	Explain how the study size was arrived at	p.4		
Quantitative variables	11	Explain how quantitative variables were handled in the analyses. If applicable, describe which groupings were chosen, and why	pp.19-21		
Statistical methods	12	(a) Describe all statistical methods, including those used to control for confounding	pp.5-6		
		(b) Describe any methods used to examine subgroups and interactions	p.6		
		(c) Explain how missing data were addressed	n/a		
		(d) *Cohort study* - If applicable, explain how loss to follow-up was addressed	n/a		
		Case-control study - If applicable, explain how matching of cases and controls was addressed			
		Cross-sectional study - If applicable, describe analytical methods taking account of sampling strategy			
		(e) Describe any sensitivity analyses	p.6		

Data access and cleaning methods		..		RECORD 12.1: Authors should describe the extent to which the investigators had access to the database population used to create the study population.<br><br>RECORD 12.2: Authors should provide information on the data cleaning methods used in the study.	pp.4,6,7<br><br>pp.4-5,19-21
Linkage		..		RECORD 12.3: State whether the study included person-level, institutional-level, or other data linkage across two or more databases. The methods of linkage and methods of linkage quality evaluation should be provided.	p.4
**Results**					
Participants	13	(a) Report the numbers of individuals at each stage of the study (*e.g.*, numbers potentially eligible, examined for eligibility, confirmed eligible, included in the study, completing follow-up, and analysed)<br>(b) Give reasons for non-participation at each stage.<br>(c) Consider use of a flow diagram		RECORD 13.1: Describe in detail the selection of the persons included in the study (*i.e.,* study population selection) including filtering based on data quality, data availability and linkage. The selection of included persons can be described in the text and/or by means of the study flow diagram.	p.4
Descriptive data	14	(a) Give characteristics of study participants (*e.g.*, demographic, clinical, social) and information on exposures and potential confounders	p.16		

		(b) Indicate the number of participants with missing data for each variable of interest<br><br>(c) *Cohort study* - summarise follow-up time (*e.g.*, average and total amount)	n/a		
Outcome data	15	*Cohort study* - Report numbers of outcome events or summary measures over time<br><br>*Case-control study* - Report numbers in each exposure category, or summary measures of exposure<br><br>*Cross-sectional study* - Report numbers of outcome events or summary measures	p.20		
Main results	16	(a) Give unadjusted estimates and, if applicable, confounder-adjusted estimates and their precision (e.g., 95% confidence interval). Make clear which confounders were adjusted for and why they were included<br><br>(b) Report category boundaries when continuous variables were categorized<br><br>(c) If relevant, consider translating estimates of relative risk into absolute risk for a meaningful time period	p.17-18,37		
Other analyses	17	Report other analyses done—e.g., analyses of subgroups and interactions, and sensitivity analyses	p.23-36		

**Discussion**					
Key results	18	Summarise key results with reference to study objectives	p.9-10		
Limitations	19	Discuss limitations of the study, taking into account sources of potential bias or imprecision. Discuss both direction and magnitude of any potential bias	p.9-10	RECORD 19.1: Discuss the implications of using data that were not created or collected to answer the specific research question(s). Include discussion of misclassification bias, unmeasured confounding, missing data, and changing eligibility over time, as they pertain to the study being reported.	pp.9-10
Interpretation	20	Give a cautious overall interpretation of results considering objectives, limitations, multiplicity of analyses, results from similar studies, and other relevant evidence	p.10		
Generalisability	21	Discuss the generalisability (external validity) of the study results	p.9-10		
**Other Information**					
Funding	22	Give the source of funding and the role of the funders for the present study and, if applicable, for the original study on which the present article is based	p.1		
Accessibility of protocol, raw data, and programming code		..		RECORD 22.1: Authors should provide information on how to access any supplemental information such as the study protocol, raw data, or programming code.	p.7

*Reference: Benchimol EI, Smeeth L, Guttmann A, Harron K, Moher D, Petersen I, Sørensen HT, von Elm E, Langan SM, the RECORD Working Committee. The REporting of studies Conducted using Observational Routinely-collected health Data (RECORD) Statement. *PLoS Medicine* 2015; in press.

*Checklist is protected under Creative Commons Attribution (CC BY) license.
